# Effects of hydraulically disconnecting consumer pumps in an intermittent water supply

**DOI:** 10.1016/j.wroa.2021.100107

**Published:** 2021-06-18

**Authors:** David D.J. Meyer, J. Khari, Andrew J. Whittle, Alexander H. Slocum

**Affiliations:** a Mechanical Engineering, MIT, 77 Massachusetts Ave., Cambridge, MA, 02139, USA; b Anonymous Partner Utility, Delhi, India; c Civil and Environmental Engineering, MIT, 77 Massachusetts Ave., Cambridge, MA, 02139, USA

**Keywords:** Intermittent water supplies (IWS), Contaminant intrusion, Minimum pressure head, Suction pumps, Water distribution network (WDN), Booster pumps

## Abstract

We estimate 250 million people receive water using private pumps connected directly to intermittently pressurized distribution networks. Yet no previous studies have quantified the presumed effects of these pumps. In this paper, we investigate the effects of installing pressure-sustaining valves at consumer connections. These valves mimic pump disconnection by restricting flow. Installing these valves during the dry season at 94% of connections in an affluent neighborhood in Delhi, India, cut the prevalence of samples with turbidity > 4 NTU by two thirds. But considering the poor reputation of pumps, installed valves had surprisingly small average effects on turbidity (-8%; *p*<0.01) and free chlorine (+0.05 mg/L; *p*<0.001; *N* = 1,031). These effects were much smaller than the high variability in water quality supplied to both control and valve-installed neighborhoods. Site-specific responses to this variability could have confounded our results. At the study site, installed valves increased network pressure during 88% of the typical supply window; valves had a maximum pressure effect of +0.62 m (95% CI [0.54, 0.71]; a 40% increase vs. control). Further research is needed to generalize beyond our study site. Nevertheless, this paper provides unique evidence showing how the deployed valves mitigated pump effects, increased network pressure and improved water safety.

## Highlights

• ~250 million people use private pumps to get water from intermittent systems.
• Installed valves mimicked pump disconnection, showing pump effects in Delhi, India.
• Valves reduced the mean turbidity by 8% and the prevalence of samples >4 NTU by 68%.
• System variability influenced mean water quality more than the valves in our study.
• Mitigating private pump effects increased pressure, improving water safety.

## Graphical abstract

**Figure:**
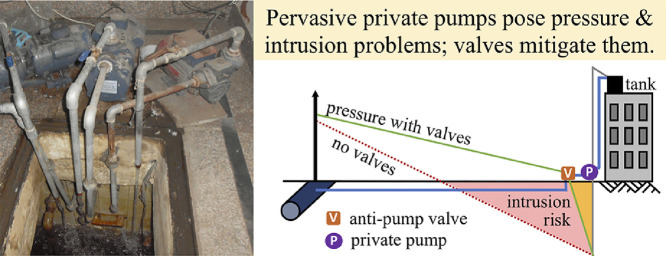

## 1 Introduction

Global estimates suggest that 21% of piped water consumers (almost 1 billion people) access water from distribution networks that are not continuously pressurized (Bivins et al., 2017; UNICEF and WHO, 2019). Contaminants can intrude into these intermittent water supplies (IWS) through compromised pipes during periods of depressurization and during periods of low-pressure water supply (Bautista-de los Santos et al., 2019; Bivins et al., 2021; Erickson et al., 2017; Kumpel and Nelson, 2016; Taylor et al., 2018b). Low distribution pressure is common in IWS, in part because these systems deliver water over short supply windows, increasing flow rates and pressure losses (Klingel, 2012). Low distribution pressure, and the associated risk of contaminant intrusion, may also result from consumer adaptations to IWS.

Consumers adapt to intermittent water availability by storing water locally (Galaitsi et al., 2016; Kumpel et al., 2017). Overhead storage further increases convenience, but typically requires consumers to use a pump (Guragai et al., 2018; Guragai et. al 2017; Kumpel et al., 2017). While some consumers pump from passively-filled, lower storage tanks, many get additional volumes of water by pumping directly from the distribution system. These directly connected “*suction pumps*” are owned and operated by consumers and are widely presumed to lower distribution pressure, promote contaminant intrusion, and reduce water volumes delivered to consumers without pumps (Ahmed, 2008; Klingel, 2012; Zérah, 2000). Suction pumps may also reduce equity in IWS as pump use varies by affluence (Zérah, 2000). Where suction pumps are common, some utilities blame consumers and their pumps for deficiencies in the water supply (e.g. Bagga (2012)).

We identified five case studies in the literature where the prevalence of suction pumps was quantified (Ahmed, 2008, Klingel, 2010, Kumpel et al., 2017, Mastaller and Klingel, 2018, Onda, 2014), four of which were based in South Asia (Table S1). These studies combine to suggest that 25% of consumers in IWS (extrapolates to 250 million people) access their water supply through a suction pump (Table S1). If the presumed effects of pumps are true, pumps pose a pervasive threat to water quality and equity, while water utilities and regulators ought to prioritize mitigating their effects.

Yet no prior research has measured the collective effects of suction pumps on water quality or pressure in any distribution system. This absence of data likely reflects the physical and political difficulty of prohibiting or preventing consumers from using their own pumps (Ahmed, 2008; Anand, 2011, 2012). To overcome such difficulties, Taylor and Slocum (2015) invented and validated the Anti-Pump Valve (APV). The APV regulates pressure and flow to mimic the existence of a storage tank upstream of the consumer's pump, thereby eliminating the suction pump's effects on the network (Taylor, 2014). Taylor (2014) used the APV to verify that individual suction pumps can cause inward pressure gradients locally (measured just upstream of the suction pump; Text S1 and Fig. S1). However, the local effects of individual suction pumps likely differ from the collective effects of many suction pumps within a network.

*This paper's first aim is to estimate the collective effects of suction pumps* in a neighborhood in Delhi, India, by observing the effects of widespread APV installation. To do so, we measured operational parameters indicative of intrusion. Since intrusion requires an inward pressure gradient, a pathway for transport, and nearby contaminants (Besner et al., 2011; Lindley and Buchberger, 2002), we also considered water pressure as surrogate indicator of water safety, i.e., lower pressure increases the likelihood of an inward pressure gradient with the concomitant risk of intrusion. In the event that suction pumps collectively produce substantial and undesirable effects, *the paper's second aim is to determine the feasibility of mitigating suction-pump effects through APV installation*.

## 2 Materials and methods

### 2.1 Hydraulic disconnection of suction pumps

**Fig. 1:**
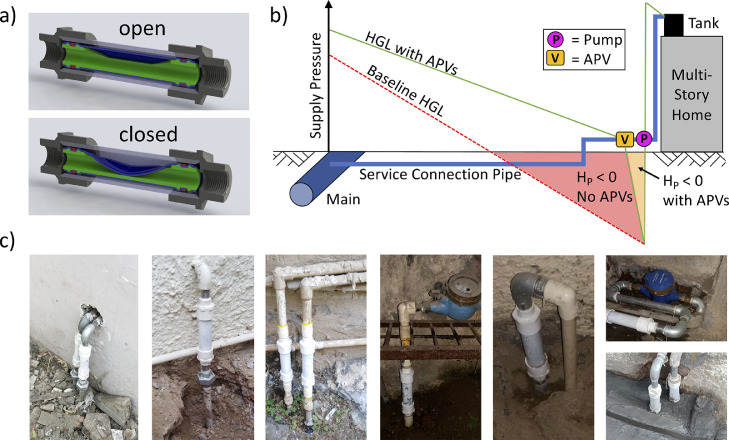
APV design, theory and installation. a) Cross sections of the APV with its membrane in the open (top) and closed (bottom) position. b) Expected hydraulic grade lines (HGL) leading to household connections with and without APVs throughout a neighborhood. c) Select photos of the white, plastic APVs installed at household connections. APV: anti-pump valve; H_P_: pressure head.

The APV is a pressure-sustaining valve with an upstream setpoint of atmospheric pressure (Fig. 1a); it was invented to throttle through flow in order to maintain positive, upstream water pressure (Fig. 1b) (Taylor and Slocum, 2015). Unlike other pressure-sustaining valves, however, the APV poses no obstruction to the flow cross-section when in its open position, thereby minimizing its reduction of pressure when open (Fig. 1a). The flow rate that consumers receive with an APV installed, even while using a suction pump, is intended to be equivalent to the flow rate they would receive with a ground-level tank filling passively (i.e. without a suction pump). The installation of an APV is therefore equivalent to “*hydraulically disconnecting*” a suction pump. Several different versions of *prototype APVs* (handmade in the U.S.) were tested at individual household connections in Delhi as part of previous work (Taylor, 2014), but APVs have not been widely deployed outside this study. For the present study, 500 plastic APVs were injection molded in the U.S. and assembled in India.

Prior testing (Taylor, 2014) suggested prototype APVs did not prevent all negative pressure (measured immediately upstream of the device), but these prototype APVs did reduce the time during which pressure head, H_p_ < −0.5 m by 80% (*p*<0.01; Fig. S1) and the time during which H_p_ < −1 m by 96% (*p*<0.001; Fig. S1). This increase in pressure along individual service connection pipes (Fig. 1b) likely reduced the local risk of contaminant intrusion. Based on these results, the cumulative effects of APVs (if installed at most connections in a neighborhood) were expected to include increasing the supply pressure in nearby water mains due to the overall reduction in flow rates and their concomitant pressure losses (e.g. ‘Main’ in Fig. 1b).

The effects of pumps and APVs depend on the supply pressure in the water main to which a consumer connects (Fig. 1b). APVs severely limit flow rates to consumers in locations where the pressure head in the water main, H_p_ < 2 m (averaged while water is being supplied); APVs are not recommended in such areas. In areas where H_p_ > 10 m, consumer pumps are unlikely to induce negative pressures and APVs would have little or no effect. Therefore, we selected a study site with 2 m ≤ H_p_ ≤ 10 m, while water was being supplied. While pressure varies spatially and temporally in IWS, 79% of Indian water utilities who reported an average pressure at consumer connections (*N* = 19) reported 2 m ≤ H_p_ ≤ 10 m (ADB and MoUD, 2007). Similarly, pressure at consumer taps in Nagpur, India was measured in this range for 83% and 71% of intermittently and continuously supplied households, respectively (Bivins et al., 2021).

### 2.2 Study design and site

We conducted the study in Delhi, India, where the public water utility had claimed that consumer pumps were their biggest problem and responsible for 90% of water contamination issues (Bagga, 2012). Budget constraints limited our study to installing APVs in a single neighborhood and so we used a Before-After, Control-Impact experimental design (BACI) (Stewart-Oaten and Bence, 2001).

During the Before Period (October 1st to November 19th, 2018), consumers in a Control Zone and an Impact Zone used their suction pumps without obstruction. Next, APVs were installed at 94% of service connections in the Impact Zone (total of 301 APVs). APVs were installed upstream of consumer pumps (often just outside consumer premises, Fig. 1c), but were not installed at 6% of connections in the Impact Zone due to lack of consumer availability, interest, and/or accessibility. Finally, both zones were observed during the After Period (December 2nd, 2018 to January 19th, 2019).

The study was conducted during the first half of Delhi's dry season: i) to minimize the variability in intrusion associated with rain events; and ii) to avoid severe water shortages sometimes associated with the latter half of the dry season. Daily rainfall during Before and After Periods was minimal, totaling 5.9 mm and 6.3 mm, respectively (Rainfall Monitoring Unit, 2020).

At the study site, the water supply was (usually) predictably intermittent (Galaitsi et al., 2016), lasting 2.5–4 h daily. Pumped water supply typically began from the underground supply reservoir at 5 am, reaching the Control and Impact Zones approximately 15 min later. The duration of the water supply window depended on reservoir levels and ended near 8 am most days during the Before Period. During the After Period, the system depressurized (briefly) at 8 am as supply valves and pumps were reconfigured and then water was often supplied for an additional 15–60 min between 8 and 9 am. When extra water was available (occasionally), the utility would extend the morning supply and/or pump water for a brief period in the afternoon or night.

The Impact Zone was selected because of its size, hydraulic isolation, high-trust relationship between consumers and the utility, history of occasionally-severe contamination, and average pressure head (between 2 and 10 m during supply times). It had 124 structures and 320 connections to the distribution system (Fig. S2b). To account for temporal variations in water quality and pressure, a nearby, similarly isolated neighborhood with 60 structures and 181 connections was selected as the Control Zone (Fig. S2a). A schematic of each zone and their relative locations in the network is included as Fig. S2.

### 2.3 Measuring water quality

At our study site, pre-study testing of water quality (methods and results in Text S2) detected fecal indicator bacteria only once in 123 samples of 100 mL (Fig. S3), plausibly due to high levels of residual free chlorine (90% > 0.5 mg/L and 31% > 1 mg/L; Fig. S3). Given the low baseline prevalence of fecal indicator bacteria at our study site, detecting a significant decrease in their prevalence would have been prohibitively expensive (Taylor et al., 2018a). More generally, detecting an intrusion event by sampling *E. coli* is unlikely in a chlorinated water supply unless samples are taken close to the source while intrusion is occurring (Hatam et al., 2020).

Aerobic endospores have been suggested as an alternative indicator of intrusion in other contexts (Cartier et al., 2009). Near our study site, aerobic endospores were consistently detected in soils (Fig. S4), but their variable presence in the treated source water (Fig. S5) discouraged their use as an indicator of intrusion. In lieu of microbial indicators, turbidity, total chlorine, and free chlorine were used as operational indicators to quantify potential changes in intrusion and water quality (Helbling and VanBriesen, 2008; WHO 2014, WHO 2017). While total chlorine was measured and modelled (Text S3), it is omitted here because it closely resembled free chlorine.

Grab samples were collected repeatedly from a convenience sample of 20 connections per zone (10 samples per zone per day, 6 days per week; approximate sampling locations in Fig. S2). Samples were collected downstream of consumers’ suction pumps but upstream of any storage tanks. Samples were immediately tested for turbidity using HACH 2100Q portable meters (0–40 NTU range; accuracy 2% of reading and stray light effects < 0.02 NTU). Samples were also promptly tested for chlorine residual (total and free) using HACH Pocket Colorimeter II MR/HR portable meters with ‘mid-range’ reagents (measurement range: 0.05–4.00 mg/L). Due to limited water and consumer availability, not all connections were sampled when scheduled. Data analysis only includes samples from connections with ≥30 observations and from days with ≥8 observations in each zone. The analyzed data includes 504 samples from 19 connections in the Control Zone and 527 samples from 20 connections in the Impact Zone. Included samples were taken on 27 days during the Before Period (524 samples), and 26 days during the After Period (507 samples).

### 2.4 Measuring pressure

During a typical intermittent water supply cycle at the study site, consumers gradually become satisfied (i.e. receive as much water as they demand (Taylor et al., 2019)), causing flow rates to decrease and pressures to increase. We hypothesized that suction pumps increased flow rates at the beginning of the supply cycle and thereby magnified the pressure differential between the start and end of supply. If true, APVs would increase the pressure (in the distribution network) at the start of supply.

Pressure in the water distribution mains was sampled at 5-minute intervals at two interior locations in each zone using Mirocom Nemos N200+ pressure loggers (layout in Fig. S2). Battery capacity, cell connectivity, and operational constraints limited the pressure coverage to 43 days with at least one pressure logger in each zone before and 43 days after installation of the APVs.

### 2.5 Data analysis

The effects of APVs were estimated using data from the Before and After Periods in the Control and Impact Zones. The intervention effects in a BACI experiment are not directly measured; instead they are found by measuring how the difference between Control and Impact Zones changes from the Before to the After Period (a difference of difference). BACI experimental designs can control for location effects and time effects, but not time effects which differ between locations.

Mean APV effects were computed using two different methods:

1 First, by computing the mean difference-of-difference between Control and Impact Zones during Before and After Periods. Uncertainty in the resultant APV-effect estimate is summarized by a 95% confidence interval generated from the t-distribution and by comparing zonal differences (Impact – Control) during the Before and After Periods.
2 Second, by using multiple linear regression with fixed effects to control for location and time effects, as is appropriate for BACI designs (Stewart-Oaten and Bence, 2001). Non-normality in the data and residuals were accounted for using bootstrapping methods appropriate for regression (stratified, case bootstrapping (Chernick, 2008)) with 9999 replications. The uncertainty in regression results was summarized by bias-corrected, accelerated, 95th-percentile intervals (Fox and Weisberg, 2019), hereafter referred to as confidence intervals (CI) for convenience.

#### 2.5.1 Water quality effects

In the first method, the average difference in free chlorine between Impact and Control Zones was computed each day, with a t-distribution-derived 95% confidence interval. The average difference between these daily differences (After – Before) was attributed to the APV's installation, also with a t-distribution-derived 95% confidence interval. Turbidity measurements were right-skewed, which we mitigated by log-transforming turbidity values before analysis (Figure S6). Log-transformed turbidity values were analyzed with the same method as free chlorine values (difference of difference with t-distribution-derived confidence intervals). Estimates of the APV's effect on ln(Turbidity) are reported in the text as equivalent percent changes; for example, if the APV's effect ΔΔln(Turbidity)=0.15, we would report APVs were associated with a 16% increase in turbidity $\left(e^{0.15}=1.16=+16\%\right)$.

In the second method, multiple linear regression estimated free chlorine (Model 2) and log-turbidity (Model 1) each day *d* at each sampled connection *c,* accounting for the presence or absence of the APV. Connection and date were treated as fixed effects, whose estimates are not generalizable and therefore are not reported in the main text. The model of free chlorine additionally included turbidity $(T_{d,c}$, not log-transformed) as a predictor.

(Model 1) $$\text{ln}\left(T_{d,c}\right)=b_{0}+b_{D,d}+b_{C,c}+b_{1}X_{APV}$$

(Model 2) $$Cl_{d,c}=b_{0}+b_{D,d}+b_{C,c}+b_{1}X_{APV}+b_{2}T_{d,c}$$

Where: $T_{d,c}$ and $Cl_{d,c}\;$ represent the estimated turbidity and free chlorine on day *d* at connection *c;* d∈{1,…,53} days; c∈{1,…,39} connections; and $X_{APV}\in \left\{0,1\right\}$, where $X_{APV}=1$ indicates that the APVs were installed in that connection's zone on that day. The effect estimates $\left\{b_{0},b_{D,d},b_{C,c},b_{1},b_{2}\right\}$ were estimated separately for Models 1 and 2. The intercepts $b_{0}$ were calculated by assuming that $b_{C,1}=b_{D,1}=0$. If APVs improved (i.e. reduced) overall turbidity, $b_{1}$ would be negative in Model 1. Conversely, if APVs improved (i.e. increased) overall concentrations of free chlorine, $b_{1}$ would be positive in Model 2.

Additionally, APV-induced changes in the distribution (rather than the mean) of water quality parameters were investigated graphically using the observed empirical cumulative distributions functions (ECDFs) from both zones during both periods. To bound the true cumulative distribution function within a confidence band around the ECDF with 95% probability, we used the Dvoretzky–Kiefer–Wolfowitz inequality (Massart, 1990).

#### 2.5.2 Pressure effect estimates

To control for variations in supply during the study, we studied the difference between the spatially averaged pressure head in the Control and Impact Zones. This pressure difference, $\Delta H_{P}$, was strongly dependent on the time of day, *t*; note *t* is not ‘date and time’ but ‘time of day’. For clarity, we focus on the window during which water was typically supplied (5:00 to 9:10 am).

In the first method, we computed $\Delta H_{P}\left(t\right)$ each day as the difference between $H_{P}\left(t\right)$ in each zone (Impact – Control). The effect of the APV was then computed as the change in $\Delta H_{P}\left(t\right)$ between periods (After – Before; a difference of difference, $\Delta \Delta H_{P}\left(t\right)$), with a t-distribution-derived 95% confidence interval.

In the second method, we estimated $\Delta H_{P}\left(t\right)$ using multiple linear regression, accounting for baseline differences in time-of-day with fixed effects. Within this regression model, the effect of the APV was estimated separately for each time of day (*t*). The final model of the pressure difference between zones was:

(Model 3) $$\Delta H_{P}\left(t\right)=b_{0}+b_{T_{1},t}+b_{T_{2},t}X_{APV}$$

Where $X_{APV}\in \left\{0,1\right\}$, where $X_{APV}=1\;$ indicates that APVs were installed. While we focus on 5:00 to 9:10 am, valve effects were estimated throughout the day (Fig. S7); ‘time of day’ was in five-minute increments *t* ∈ {00:00, 00:00, . . . , 23:55}. Together, $b_{0}+b_{T_{1},t}$ estimate how $\Delta H_{P}\left(t\right)$ varied during an average day in the Before Period. Each $b_{T_{2},t}$ (e.g. $b_{T_{2},05:20}$) estimates how $\Delta H_{P}$ changed at that time of day (*t*) during the After Period (e.g. at 05:20 am); this change was then attributed to APVs. The constant $b_{0}$ was calculated by assuming that $b_{T_{1},00:00}=0$.

## 3 Results and discussion

The transition between periods unfortunately coincided with a change in the supply pattern; on most days in the After Period, the utility supplied water for longer and with lower pressure than before. The BACI experimental design accounts for these changes, provided they would have affected both zones equally had there been no intervention. If these changes would have affected zones differently, that difference would (erroneously) be attributed to APVs.

### 3.1 APV effects on water quality

**Fig. 2:**
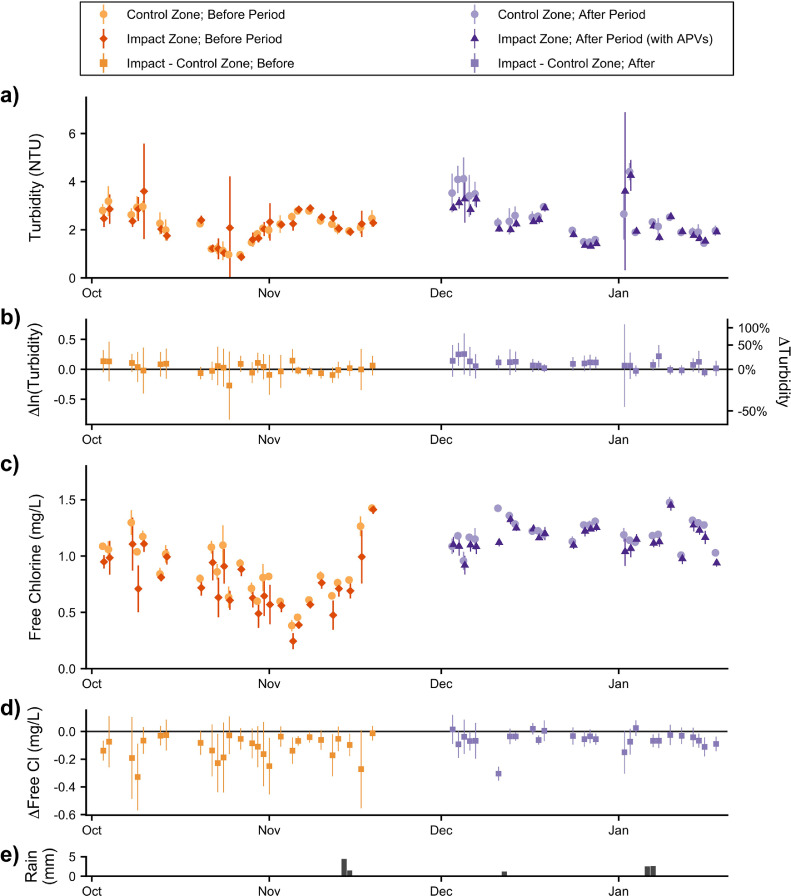
Water quality variations and zonal differences. Substantial water quality variations in the mean turbidity (a), and free chlorine (c) were observed in the Control Zone (lighter dots) and Impact Zone (darker diamonds and triangles). Daily variations in water quality between zones were controlled for by computing each day's difference (Impact – Control) in mean free chlorine (d) and log-mean turbidity (b). Rainfall was infrequent and minimal during both periods (e). Error bars indicate t-distribution-derived 95% confidence intervals.

**Fig. 3:**
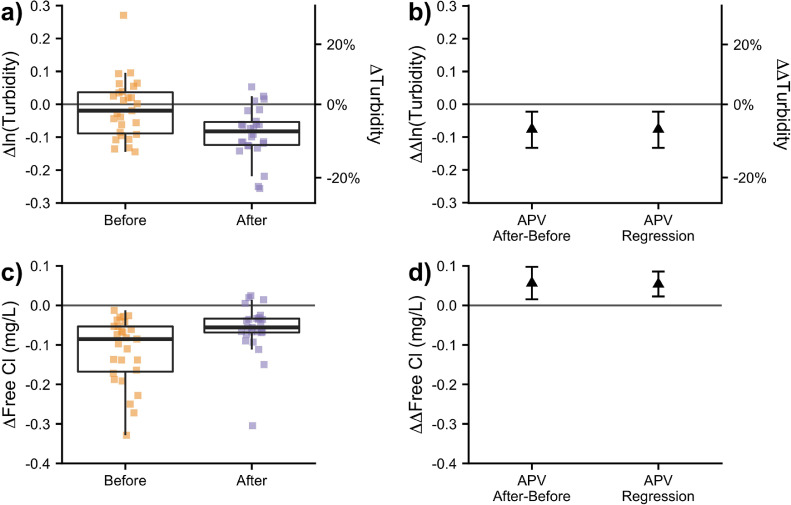
APV effects on water quality. The difference each day between zones (Impact – Control) in log-mean turbidity (a) and mean free chlorine (c) varied between Before and After Periods. The effect of APV installation is reported as the average difference (After – Before) in these differences for log-turbidity (b) and free chlorine (d). These estimates agree well with the regression models of APV impact (Models 1 and 2) (b, d). APV installation caused statistically, significant but modest improvements in water quality, which were much smaller than the baseline variability in water quality. Boxplots (a and c) depict median, interquartile range and extent of data excluding outliers. Error bars (b and d) indicate 95% confidence intervals.

Throughout the Before and After Periods, substantial variations in mean water quality were observed, even in the Control Zone (Fig. 2). Amidst these large temporal variations in quality parameters, the installation of APVs modestly improved tracked indicators. The difference-of-differences method suggested APVs reduced mean turbidity by 7% (95% confidence interval (CI) [2%, 12%]; Fig. 3b) and increased free chlorine by 0.05 mg/L (95% CI [0.02, 0.09]; Fig. 3d). Similarly, Regression Models 1 and 2 suggest that APVs changed the turbidity and free chlorine by a mean of −8% (*p*<0.01) and +0.05 mg/L (*p*<0.001), respectively (Table 1 and Figs. 3b and 3d). In both methods, variations in water quality associated with recent rainfall were controlled for by analyzing the differences between zones on a daily basis.

**Table 1:** Regression-computed effect of APVs on turbidity and free chlorine in grab samples.

Effect Estimates & Goodness of Fit	Dependent Variables	
	ln(Turbidity) Model 1	Free Chlorine (mg/L) Model 2
Constant (*b_0_*)	0.96^⁎⁎⁎^ (0.89, 1.04)	1.56^⁎⁎⁎^ (1.50, 1.62)
APV (*b_1_*)	−0.08^⁎⁎^ (−0.13, −0.02)	0.05^⁎⁎⁎^ (0.02, 0.09)
Turbidity (*b_2_*)		−0.03^⁎⁎^ (−0.04, −0.02)
Dates (*b_D,2_*-*b_D,53_*)	df=52; *F* = 38^⁎⁎⁎^	df=52; *F* = 66^⁎⁎⁎^
Connections (*b_C,_*_2_-b_C_*_,39_*)	df=38; *F* = 0.9	df=38; *F* = 4.8^⁎⁎⁎^

Observations	1031	1031
Adjusted R^2^	0.66	0.83
F Statistic	22^⁎⁎⁎^ (df = 91; 939)	57^⁎⁎⁎^ (df = 92; 938)

*p<0.05, **p<0.01, ***p<0.001. Bracketed terms following effect estimates are the 95% bias-corrected, accelerated percentile intervals for each effect estimate. The significance of Date and Connection factors are summarized by the F-Statistic (from Type II ANOVA); their individual estimates are included in Table S2. Turbidity is measured in NTU. Regression Models 1 and 2 estimate ln(turbidity) and free chlorine, respectively. df: degrees of freedom; APV: Anti-Pump Valve.

Improvements in log-mean turbidity and mean free chlorine due to APVs, while statistically significant, were close to the precision of the measurement methods and were more than an order of magnitude smaller than the observed temporal variations in these parameters (Figs. 2 and 3), due presumably to fluctuations in treated water quality. The limited improvements in overall water quality were particularly unexpected given how frequently pumps are purported to degrade water quality (including in Delhi). The limited water quality effects may have resulted in part from selecting zones with$\;H_{P}\geq$ 2 m.

**Fig. 4:**
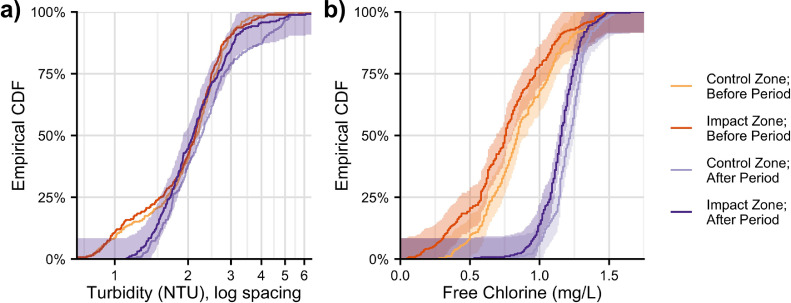
Empirical Cumulative Distribution Function (CDF) of turbidity and free chlorine. Lines show the distributions of measured of turbidity (a) and free chlorine (b) taken during the Before (yellow-orange) and After (purple) Periods from the Control (light) and Impact (darker) Zones. Turbidity values > 6.5 NTU (< 1% of values) are omitted for clarity. The 95% confidence band is shaded around the distribution of turbidity in the Impact Zone during the After Period (a) and around each distribution of free chlorine (b).

While APVs were associated with a small overall reduction in turbidity (~8%), their presence during the After Period was also associated with a 68% reduction (*p*<0.001) in the prevalence of samples with turbidity > 4 NTU (from 13% to 4.2%; Fig. 4a). Samples with turbidity > 4 NTU increased during the After Period in both zones, but the increase was much more pronounced in the Control Zone (6x larger; Fig. 4a). Nevertheless, with APVs installed, 4.2% of samples in the Impact Zone had turbidity > 4 NTU (Fig. 4a) and the highest measured value of turbidity (18 NTU) occurred at a connection with an APV installed.

The increase in high turbidity (> 4 NTU) in the Control Zone could be explained by: i) a localized turbidity-increasing phenomenon in the Control Zone during the After Period; ii) a phenomenon that would have increased the prevalence of high turbidity in both zones, but the phenomenon was mitigated in the Impact Zone by APVs; or iii) a combination of i) and ii). The experimental design does not allow us to distinguish between these possibilities. In any case, APVs did not eliminate all instances of high turbidity.

Increases in turbidity can indicate upstream contaminant intrusion, backflow, and/or detachment of the biofilm or rust (Husband and Boxall, 2011; WHO, 2017, 2014). The observed overall reductions in turbidity and especially in high-turbidity events associated with APVs suggest that suction pumps do promote or exacerbate turbidity-increasing phenomena, posing a threat to water quality. The relationship between health and turbidity in distribution networks continues to be debated (WHO, 2017); nevertheless, measured turbidity was sometimes high enough (Figs. 2a and 4a) to increase chlorine demand and possibly to limit the disinfection potential of residual chlorine (LeChevallier et al., 1981; WHO, 2017).

During the Before Period, free chlorine was < 0.5 mg/L in 7.8% and 19% of samples in the Control and Impact Zones (Fig. 4b). During the After Period, however, free chlorine was always > 0.5 mg/L (Fig. 4b). Substantial increases free chlorine in both zones suggest a change in upstream treatment processes rather than a change associated with APV installation.

Limited APV-induced improvements in water quality do not necessarily imply limited improvements in intrusion risk or water safety since contaminant intrusion requires the presence of a contaminant in the vicinity of a transport pathway and an inward pressure gradient (Besner et al., 2011; Lindley and Buchberger, 2002). Our study was conducted during the dry season (Fig. 2e), when contamination events are less frequent (Kumpel and Nelson, 2013), and in an affluent and residential area with fewer contaminants near water pipes than may be expected in informal settlements. In a different season or at a different site, pump-induced contaminant intrusion may have been more prevalent. Further research is needed to generalize the current findings.

### 3.2 APV effects on pressure

To make a more generalizable assessment of the risk of intrusion, the prevalence and magnitude of low pressure is reported. Lower pressure increases the likelihood of an inward hydraulic gradient and the associated risk of contaminant intrusion (Besner et al., 2011; Lindley and Buchberger, 2002) and hence, provides a surrogate indicator of reduced water safety.

**Fig. 5:**
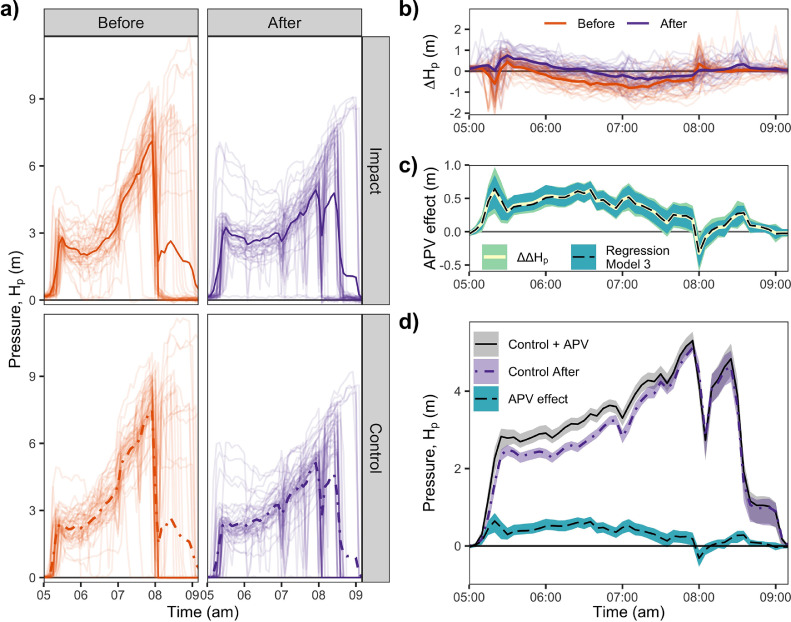
Measured pressure profiles and Anti-Pump Valve (APV) effect estimates. a) The average pressure vs. time of day for the Impact (solid line) and Control (dash-dot line) Zones varied between Before (orange) and After (purple) Periods during the typical water supply timing, 5:00 to 9:10 am. Each day's pressure trace is also shown with partial transparency. b) Measured pressure differences ($\Delta H_{P}$) between zones (Impact – Control) each day (partially transparent) and averaged during Before and After Periods (thick lines). c) APV effect was estimated as the mean difference in measured $\Delta H_{P}$$(\Delta \Delta H_{P}$; yellow solid line) and by regression Model 3 (black dashed line); 95% confidence intervals were generated using the t-distribution (light green band) and with bootstrapped regression (darker teal). d) The potential impact of APV installation: regression-estimated APV effects (dashed line with shaded teal confidence band) superimposed on the measured pressure profile in the Control Zone during the After Period (purple dash-dot line with shaded confidence band), showing how APV effects would have changed pressure in the Control Zone (Control + APV curve; solid black line with gray shaded confidence interval). APV: Anti-pump valve.

The pressure difference between zones (Impact – Control; $\Delta H_{P}\left(t\right)$) varied with the time of day and changed when APVs were installed (Fig. 5a). The effect of APVs was first computed as the mean difference in $\Delta H_{P}\left(t\right)$ ($\Delta \Delta H_{P}\left(t\right)$; Fig. 5b) and second with regression Model 3. Estimates from both methods and their 95% confidence ranges match very closely (Fig. 5c). Both methods suggest APV effects varied with time of day (Fig. 5c). As the regression model more robustly accounted for non-normality, we focus on describing its numerical estimates in the text. To contextualize the magnitude and timing of APV effects, the regression-estimated APV effects (Fig. 5c and Table S3) were superimposed on the mean pressure profile observed in the Control Zone during the After Period (Fig. 5a), resulting in Fig. 5d.

As expected, APVs increased pressure in the distribution network, especially at the start of supply. At 5:20 am, APVs had their maximum effect on pressure, increasing it by a mean of 0.62 m (95% CI [0.54, 0.71]; Fig. 5c), equivalent to increasing pressure in the Control Zone by 40% (Fig. 5d). During the period from 5:15 to 7:00 am, which begins when water typically reached the Control and Impact Zones, APVs increased the pressure by a mean of 0.48 m (equivalent to a 21% pressure increase in the Control Zone pressure; Fig. 5d). From 7:05 to 7:55 am, APVs increased pressure by a mean 0.29 m (equivalent to an 8% increase in the Control Zone; Fig. 5d). Finally, from 8 to 9:05 am, the mean pressure increase was negligible (0.05 m, or 2% in the Control Zone; Fig. 5d). At 8:00 am, the computed APV effect was negative (−0.32 m 95% CI [−0.60, −0.04]; Fig. 5c). APVs in the Impact Zone slowed flow rates; we hypothesize this left more consumers withdrawing water and deflating pressure (compared to the Control Zone) during the temporary depressurization at 8:00 am during the After Period (Fig. 5a). Overall, APVs increased pressure 88% of the time from 5:00 to 9:10 am, but their efficacy decreased during second half of the supply window when many consumers had received enough water, reducing the number of active pumps (Fig. 5d).

Since many common contaminant sources have low pressure (e.g. adjacent saturated soils), even modest pressure increases in pipes with low pressure can meaningfully reduce the prevalence and/or severity of inward pressure gradients, thereby reducing the risk and/or severity of contaminant intrusion while water is being supplied (Besner et al., 2011; Collins and Boxall, 2013; Ebacher et al., 2012; Taylor et al., 2018b). More research is needed to understand how low pressures affect water safety in intermittent water supplies. As part of its water safety plans, The World Health Organization recommends the removal of contaminants in the vicinity of pipes in IWS (WHO, 2014); this recommendation should certainly be emphasized wherever consumers use suction pumps.

Consumers who could afford pumps and household water treatment often expressed that they wanted more water, not water of better quality. Yet allowing suction pumps to deliver more water to pump users, while potentially compromising water quality, harms consumers who cannot afford to both pump and treat their water. APVs reduced this suction-pump-induced inequity by increasing pressure and disrupting the hydraulic connection between pump size and the volume of water supplied.

### 3.3 Acceptability vs. efficacy of APVs

Attempts to physically disconnect consumer pumps often encounter strong community and political resistance (e.g., see Anand (2012) or Björkman (2014)). To avoid this in the Impact Zone, we worked closely with community representatives and secured strong initial support for APVs from residents and their association. Despite selecting Impact and Control Zones with 2 m $\leq H_{P}\leq$ 10 m, after APV installation many Impact Zone consumers were concerned about flow rates and some complained. In response to each consumer complaint, the effects of the complainant's APV were reduced by moving the APV further upstream from the complainant's suction pump and/or by lowering the installed elevation of the APV.

The flow rate received by consumers is physically linked to the pressure differential between their connection and the water supply main. While suction pumps serve to increase both the pressure differential and the flow rate, APVs reduce both. APV-induced flow rate reductions were modest (via site selection and complaint response), hence our study was limited in the magnitude of observable pressure changes associated with APV installation. Our reported results do not represent the maximum technical effects that APVs could achieve, nor the maximum harm that suction pumps may cause. Instead, the results indicate the potential benefits of disconnecting consumer pumps (hydraulically or physically) in locations where disconnections are at least marginally acceptable to consumers. Pumps are likely a larger problem in areas where disconnection would be unacceptable to consumers and a lesser problem where disconnection would go uncontested. Consumer acceptance of APVs declined when consumers noticed flow rate reductions immediately after APV installation. Acceptability would likely improve if APVs were integrated into water meters or backflow preventers and installed when making new service connections or replacing existing service connections.

Given the acceptability-efficacy tradeoff of pump disconnection, utilities should continue to prioritize other system improvements such as removing contaminants near pipes and repairing leaks. At our study site, the utility should also prioritize improving the consistency of water treatment.

## 4 Limitations

This study had several notable limitations. First, the acceptability of APVs was assessed anecdotally. Future studies should seek to formalize the response of consumers to varying changes in their flow rates and delivered volumes. Second, IWS are remarkably heterogeneous and generalizing across such heterogeneity is not possible with this study, which compared two neighborhoods over 13 weeks. Third, the likelihood of contaminant intrusion during the dry season in an affluent neighborhood was low, limiting the potential of directly observing pump-induced intrusion. Fourth, our results depend on the assumption that the Control and Impact Zones would respond equally to temporal changes; yet differences between the zones were present, most notably their sizes, and the system varied substantially over time. If the zones responded differently to these temporal variations, these differences would be mistakenly attributed to APVs. A longer, multi-site study would mitigate these concerns and better quantify the potential role of APVs in improving IWS globally.

We used APVs to estimate pump effects but could only do so where APV installation was acceptable to consumers, limiting our ability to study systems severely affected by pumps. This limitation is an important qualifier of our results insofar as APV effects are used to infer the effects of suction sumps. Nevertheless, our findings are of practical importance as they suggest the potential benefits of pump mitigation, where such mitigation would be (marginally) acceptable to consumers.

## 5 Conclusions

We conclude that:

• At the study site, hydraulically disconnecting suction pumps (by installing Anti-Pump Valves) had only modest effects on mean water quality, especially when compared to variations in treated water quality, casting doubt on the applicability of the public utility's claim that 90% of contamination could be traced to suction pumps.
• The absence of a substantial association between overall water quality and pump-mitigating valves at our study site suggests utilities, researchers, and policy makers should be more cautious when making generalized claims about the effects of consumer pumps.
• Some evidence was found to suggest that, at least at the study site, mitigating suction pumps reduces the prevalence of high-turbidity-generating phenomena such as contaminant intrusion or biofilm detachment. Observed turbidity levels were sometimes high enough to impede residual disinfection, warranting further study.
• Further evidence was found suggesting that suction-pump-mitigating valves can increase water pressure, especially during the beginning of the water supply window, thereby reducing the risk of contaminant intrusion when most consumers are withdrawing water.
• Given the prevalence of suction pumps in intermittent water supplies and the regularity with which consumer pumps are assumed to cause water quality issues, additional research is needed to further specify how and where pumps are most problematic.
• As a utility improves the pressure and duration of its intermittent supply, the influence of suction pumps is expected to decrease.
• Utilities should consider the tradeoffs between the efficacy and acceptability of disconnecting suction pumps (hydraulically or physically). Other interventions such as improving treated water quality, removing nearby contaminants, and repairing leaks should remain urgent priorities.
• Upwards of 250 million people access water using suction pumps connected to intermittent water supplies. Suction pumps can worsen equity, reduce pressure and increase the risk of contaminant intrusion. At the study site, Anti-Pump Valves mitigated these effects.

## Data availability

All data supporting this study are available in an online repository, separated into three datasets:

Dataset S1: Replication data for this paper. [dataset] (Meyer et al., 2020a)

Dataset S2: Data used to initially test APVs. [dataset] (Meyer and Slocum, 2020)

Dataset S3: Water quality and soil testing results from pre-study testing. [dataset] (Meyer et al., 2020b)

## Datasets

Meyer, D., Slocum, A., 2020. Replication Data for: Taylor D (2014) Reducing booster-pump-induced contaminant intrusion in Indian water systems with a self-actuated, back-pressure regulating valve. https://doi.org/10.5683/SP2/FYIZNS.

Meyer, D., Whittle, A., Khari, J., Slocum, A., 2020a. Replication Data for: Effects of hydraulically disconnecting consumer pumps in an intermittent water supply. v1.2. https://doi.org/10.5683/SP2/HB5WXD UNF:6:PbKpIXfo/SpjicFVXYo1ug== [fileUNF].

Meyer, D., Whittle, A., Khari, J., Slocum, A., 2020b. Baseline Data Supporting: Effects of hydraulically disconnecting consumer pumps in an intermittent water supply. v2.3. https://doi.org/10.5683/SP2/PGSTAH UNF:6:GfiAxv6t0Se7IXK4dUTxHw== [fileUNF].

## Declaration of Competing Interest

The authors declare the following competing financial interests or personal relationships that could have appeared to influence the work reported in this paper: DM and AS are co-inventors of the APV and would have shared some of the patent royalties were it to have been profitably licensed; instead, the patent application has been abandoned. JK works for the utility supplying water to the Control and Impact Zones; this utility has performance incentives based on quality water. The authors declare that these interests have not substantially influenced the outcome of the work, how it has been presented, or the decision to publish.

## Appendix Supplementary materials

Image, application 1

## References

[bib0001] ADB MoUD (2007). Benchmarking and Data Book of Water Utilities in India. Asian Development Bank (ADB) and Ministry of Urban Development.

[bib0002] Ahmed N. (2008). Water Supply in Karachi: Issues and Prospects.

[bib0003] Anand N. (2012). Municipal disconnect: on abject water and its urban infrastructures. Ethnography.

[bib0004] Anand N. (2011). Pressure: the politechnics of water supply in Mumbai. Cultural Anthropol..

[bib0005] Bagga B. (2012). Water Crisis Grips Delhi, DJB Blames Residents. http://indiatoday.intoday.in/story/water-crisis-grips-delhi-djb-blames-residents/1/200100.html.

[bib0006] Bautista-de los Santos Q.M., Chavarria K.A., Nelson K.L. (2019). Understanding the impacts of intermittent supply on the drinking water microbiome. Curr. Opin. Biotechnol..

[bib0007] Besner M.-.C., Prévost M., Regli S. (2011). Assessing the public health risk of microbial intrusion events in distribution systems: conceptual model, available data, and challenges. Water Res..

[bib0008] Bivins A., Lowry S., Wankhede S., Hajare R., Murphy H.M., Borchardt M., Labhasetwar P., Brown J. (2021). Microbial water quality improvement associated with transitioning from intermittent to continuous water supply in Nagpur. India. Water Res..

[bib0009] Bivins A.W., Sumner T., Kumpel E., Howard G., Cumming O., Ross I., Nelson K., Brown J. (2017). Estimating infection risks and the global burden of diarrheal disease attributable to intermittent water supply using QMRA. Environ. Sci. Technol..

[bib0010] Björkman L. (2014). Becoming a slum: from municipal colony to illegal settlement in Liberalization-Era Mumbai. Int J Urban Reg Res.

[bib0011] Cartier C., Besner M.C., Barbeau B. (2009). Evaluating aerobic endospores as indicators of intrusion in distribution systems. J.: American Water Works Assoc..

[bib0012] Chernick M.R. (2008). Bootstrap methods: a Guide For Practitioners and researchers, Second. ed, Wiley series in Probability and Statistics.

[bib0013] Collins R., Boxall J. (2013). Influence of ground conditions on intrusion flows through apertures in distribution pipes. J. Hydraul. Eng..

[bib0014] Ebacher G., Besner M.C., Clément B., Prévost M. (2012). Sensitivity analysis of some critical factors affecting simulated intrusion volumes during a low pressure transient event in a full-scale water distribution system. Water Res..

[bib0015] Erickson J.J., Smith C.D., Goodridge A., Nelson K.L. (2017). Water quality effects of intermittent water supply in Arraiján, Panama. Water Res..

[bib0016] Fox J., Weisberg S. (2019). Bootstrapping Regression Models in R, in: An R Companion to Applied Regression.

[bib0017] Galaitsi S.E., Russell R., Bishara A., Durant J.L., Bogle J., Huber-Lee A. (2016). Intermittent domestic water supply: a critical review and analysis of causal-consequential pathways. Water (Switzerland).

[bib0018] Guragai B., Hashimoto T., Oguma K., Takizawa S. (2018). Data logger-based measurement of household water consumption and micro-component analysis of an intermittent water supply system. J. Clean. Prod..

[bib0019] Guragai B., Takizawa S., Hashimoto T., Oguma K. (2017). Effects of inequality of supply hours on consumers’ coping strategies and perceptions of intermittent water supply in Kathmandu Valley, Nepal. Sci. Total Environ..

[bib0020] Hatam F., Besner M.-.C., Ebacher G., Prévost M. (2020). Limitations of *E. coli* monitoring for confirmation of contamination in distribution systems due to intrusion under low pressure conditions in the presence of disinfectants. J. Water Resour. Plann. Manage..

[bib0021] Helbling D.E., VanBriesen J.M. (2008). Continuous monitoring of residual chlorine concentrations in response to controlled microbial intrusions in a laboratory-scale distribution system. Water Res..

[bib0022] Husband P.S., Boxall J.B. (2011). Asset deterioration and discolouration in water distribution systems. Water Res..

[bib42] Klingel P. (2010). Von intermittierender zu kontinuierlicher Wasserverteilung in Entwicklungsländern (From intermittent to continuous water distribution in developing countries) (Dr.-Ing. Dissertation).

[bib0023] Klingel P. (2012). Technical causes and impacts of intermittent water distribution. Water Sci. Technol.: Water Supply.

[bib0024] Kumpel E., Nelson K.L. (2016). Intermittent water supply: prevalence, practice, and microbial water quality. Environ. Sci. Technol..

[bib0025] Kumpel E., Nelson K.L. (2013). Comparing microbial water quality in an intermittent and continuous piped water supply. Water Res..

[bib0026] Kumpel E., Woelfle-Erskine C., Ray I., Nelson K.L. (2017). Measuring household consumption and waste in unmetered, intermittent piped water systems. Water Resour. Res..

[bib0027] LeChevallier M.W., Evans T.M., Seidler R.J. (1981). Effect of turbidity on chlorination efficiency and bacterial persistence in drinking water. Appl. Environ. Microbiol..

[bib0028] Lindley T.R., Buchberger S.G. (2002). Assessing intrusion susceptibility in distribution systems. J. Am. Water Works Assoc..

[bib0029] Massart P. (1990). The tight constant in the Dvoretzky-Kiefer-Wolfowitz Inequality. Ann. Probab..

[bib0030] Unit Rainfall Monitoring (2020). Daily rainfall data in South Delhi Oct-18 to Jan-19 (Daily rainfall data). Hydromet Division, India Meteorological Department. Ministry Earth Sci..

[bib41] Mastaller M., Klingel P. (2018). Application of a water balance adapted to intermittent water supply and flat-rate tariffs without customer metering in Tiruvannamalai, India. Water Science and Technology: Water Supply.

[bib43] Onda K.S. (2014). Intermittent vs. continuous water supply: what benefits do households actually receive? Evidence from two cities in India (Master’s Report).

[bib0031] Stewart-Oaten A., Bence J.R. (2001). Temporal and spatial variation in environmental impact assessment. Ecol. Monogr..

[bib0032] Taylor D.D.J. (2014). Reducing Booster-Pump-Induced Contaminant Intrusion in Indian water Systems With a self-actuated, Back-Pressure Regulating Valve (Thesis).

[bib0033] Taylor D.D.J., Khush R., Peletz R., Kumpel E. (2018). Efficacy of microbial sampling recommendations and practices in sub-Saharan Africa. Water Res..

[bib0034] Taylor D.D.J., Slocum A.H. (2015). Self-throttling Valves For Residential Water Supply Systems. https://patents.google.com/patent/WO2015100502A1.

[bib0035] Taylor D.D.J., Slocum A.H., Whittle A.J. (2019). Demand satisfaction as a framework for understanding intermittent water supply systems. Water Resour. Res..

[bib0036] Taylor D.D.J., Slocum A.H., Whittle A.J. (2018). Analytical scaling relations to evaluate leakage and intrusion in intermittent water supply systems. PLoS ONE.

[bib0037] UNICEF W.H.O. (2019). Progress On Household Drinking water, Sanitation and Hygiene 2000-2017. Special focus On Inequalities.

[bib0038] WHO (2017). Water Quality and Health-Review of turbidity: Information for Regulators and Water Suppliers (Technical Brief No. WHO/FWC/WSH/17.01).

[bib0039] WHO (2014). Water Safety in Distribution Systems.

[bib0040] Zérah M.-.H. (2000). Water, Unreliable Supply in Delhi.

