# Supplementary material for: Effects of hydraulically disconnecting consumer pumps in an intermittent water supply
Source: Water Res X. 2021 Jun 18;12:100107. doi: 10.1016/j.wroa.2021.100107 (PMC8319575; doi:10.1016/j.wroa.2021.100107)
Supplement: Supplementary file 1 [file mmc1.pdf]

Supplementary Materials for: Effects of hydraulically disconnecting consumer pumps in an intermittent water supply

David D. J. Meyer[a,1,*], J. Khari[b], Andrew J. Whittle[c], Alexander H. Slocum[a]

[a] *Mechanical Engineering, MIT, 77 Massachusetts Ave., Cambridge, MA, 02139*

[b] *Anonymous Partner Utility, Delhi, India*

[c] *Civil and Environmental Engineering, MIT, 77 Massachusetts Ave., Cambridge, MA, 02139*

[1] *Present Address: Civil and Mineral Engineering, University of Toronto, 55 St. George St., Toronto, Ontario, M5S 0C9*

Contents:

Text S1: A brief summary of prototype APV performance

Text S2: Methods and results from pre-study water and soil testing

Text S3: Regression model and results for total residual chlorine

Fig. S1: Prototype APV efficacy

Fig. S2: Study site layout

Fig. S3: Pre-study sampling of free chlorine and *E. coli*

Fig. S4: Concentration and correlation of aerobic endospores and *E. coli* in sampled soil

Fig. S5: Aerobic endospore concentrations in treated water before and after distribution

[*] Corresponding author. *Email address*: david.meyer@utoronto.ca

Fig. S6: Distribution of turbidity and log-turbidity values.

Fig. S7: Estimated effect APVs would have had in the Control Zone at all times

Table S1: Prevalence of consumer pumps in intermittent water supplies

Table S2: Full water quality regression models (Models 1, 2 and S1)

Table S3: Regression Model 3, estimating APV effects on pressure by time of day

Datasets cited in Texts S1-S3

References cited in Texts S1-S3

Text S1: A brief summary of prototype APV performance

In a convenience sample of 19 houses from seven neighborhoods in Delhi, India, Taylor (2014) compared pressure upstream of consumers' suction pumps with and without an APV installed. Seven of 19 households were supplied intermittently, but all used their suction pumps regularly. The average baseline (without APV) prevalence of pressure less than -1 m, measured immediately upstream of the suction pump, was 52 minutes/house/day (Baseline in Fig. S1). The average baseline duration of pressure $\leq$ 0 m was 6.4 hours/household/day (Fig. S1).

Pressure readings were paired by household and compared *with* and *without* the APV installed. The distribution of pressure was compared within all 19 pairs by randomization (i.e. randomizing the labelling of the pairs 100,000 times).

Aggregate results from tests of three prototype versions of APVs showed the APV reduced the duration of pressure head less than -0.5 m by 80% ($p<0.01$; Fig. S1) and reduced the duration of pressure head <-1 m by 96% ($p<0.001$; Fig. S1). Timeseries plots of each of these 19 households with and without the valve can be found in Taylor (2014). Replication data is available as Dataset S2 [Dataset] (Meyer and Slocum, 2020).

Text S2: Methods and results from pre-study water and soil testing

S2.1 Methods

Four isolated hydraulic zones, supplied by a common reservoir, were selected in consultation with the water utility to represent a variety of socioeconomic conditions and to include one "unauthorized zone." Two zones had household service connections that had been replaced within the previous three years; two had older service connections. Grab sample locations within zones were selected based on convenience, consumer willingness, and water availability. Consumers in the 'unauthorized zone' did not have official water connections. Instead, the water utility meters the zonal inlet and does not maintain distribution pipes or consumer water meters (though most houses do have water piped on premise).

Water quality sampling was done at the supply reservoir's outlet, at the inlet to each of the four zone and at several households in each zone, provided water was available. Household samples were taken downstream of each household's suction pump and before any storage tanks. Sampling downstream of pumps was necessary in order to have enough water pressure to collect a sample. Water samples were tested for free and total chlorine (APHA 22nd Ed. 4500 Cl-G), *E. coli* (ISO 9308 1:2000), and aerobic endospores (APHA 22nd Ed. 9218-B).

Additionally, 23 soil samples were taken from 13 locations close to water sampling points in the distribution network. Most samples were taken from soil near the surface without using a shovel (depth ranging from 2 cm to 20 cm below the surface). Two sample locations were taken from open excavations in which maintenance was being done on water distribution pipes. All soil samples were tested for *E. coli* (ISO 9308 1:2000) and aerobic endospores (APHA 22nd Ed. 9218-

B). Soil samples tests for aerobic endospores and *E. coli* had a detection limit of 100 CFU/g of soil.

All sample collection and processing (water and soil) was done by FARELABS Pvt. Ltd. Anonymized replication data is available as Dataset S3 [Dataset] (Meyer et al., 2020).

S2.2 Results

High levels of free and total chlorine were observed in most water quality samples from the reservoir outlet (97% > 0.5 mg/L; 61% > 1 mg/L; N=33; Fig. S3) and the distribution network (90% > 0.5mg/L; 31% > 1 mg/L; N=123; Fig. S3). *E. coli* was detected in only one distribution network sample (0.8%; N=123), which also had low free and total chlorine (Fig. S3).

Endospores were prevalent in all 23 soil samples gathered at 13 distinct locations. *E. coli* was present in concentrations above the detection limit in 15 of 23 soil samples. When *E. coli* was detected, its concentration was moderately correlated with aerobic endospore concentrations (Fig. S4).

Unfortunately, aerobic endospores were detected in the treated water leaving the reservoir (before distribution) in varying and substantial concentrations (Fig. S5), preventing the use of aerobic endospores as an intrusion indicator at our study site. In IWS without aerobic endospores in the treated water, they might be a useful and more chlorine-tolerant intrusion indicator.

Text S3: Regression model and results for total residual chlorine

Multiple linear regression was also used to estimate the concentration of total chlorine ($Cl_{d,c}^{total}$) on each day d at each sampled connection c, accounting for the presence or absence of the APV:

$$Cl_{d,c}^{total} = b_0 + b_{D,d} + b_{C,c} + b_1 X_{APV} + b_2 T_{d,c} \qquad \text{(Model S1)}$$

Where variables are as defined in the main text.

Regression Model S1 suggests that APVs had only a modest effect on total residual chlorine, increasing it by a mean of +0.04 mg/L ($p<0.05$; Table S2).

Supplementary figures and tables:

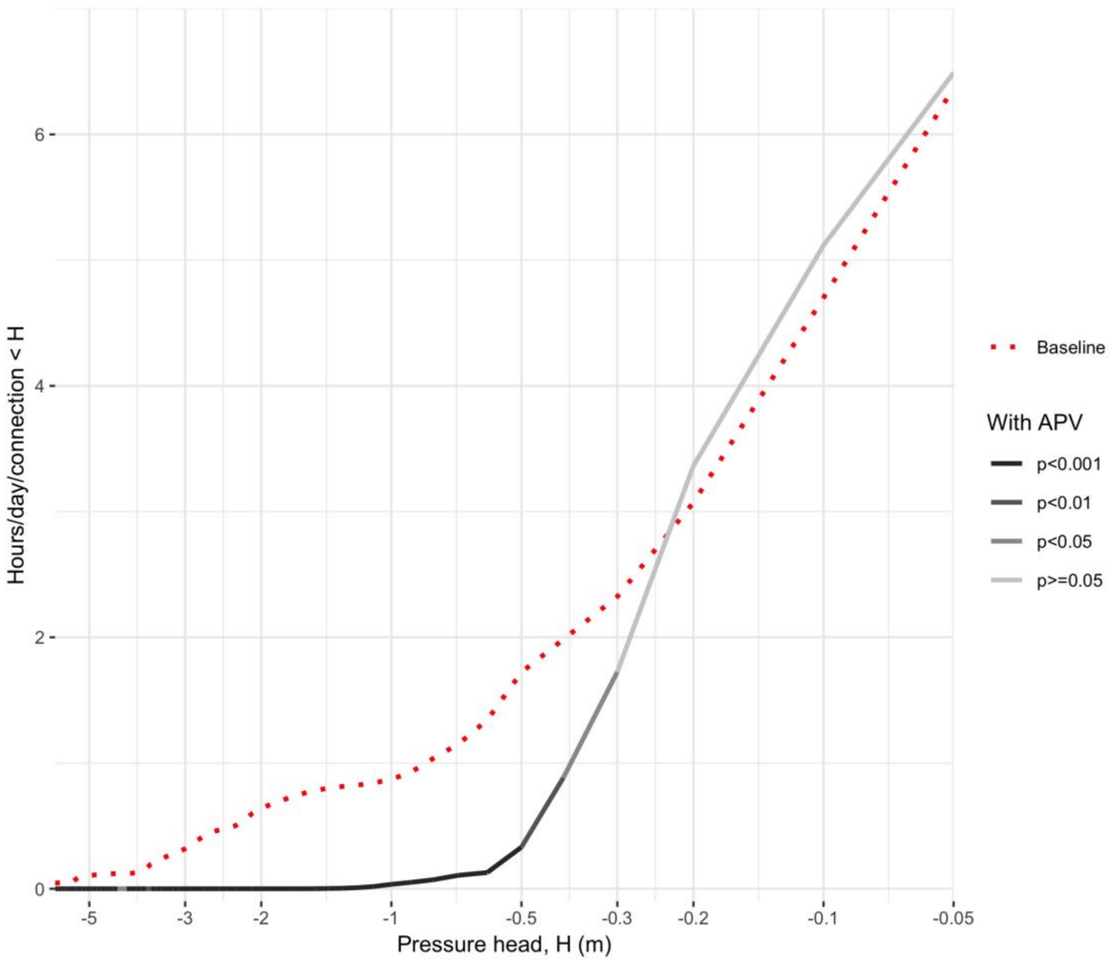

Fig. S1. Prototype APV efficacy. During previous testing of prototype APVs, the measured prevalence of negative pressure head (log spacing) reduced with an APV (solid black and grey line) compared to without an APV (red dotted line), depending on the severity of negative pressure. Baseline measurements (red dotted line) demonstrate the prevalence of negative pressure upstream of consumers' suction pumps. APVs were more effective at reducing severe negative pressure. The significance of APV effects is shown as the shading, from lightest grey (not significant) to black ($p<0.001$).

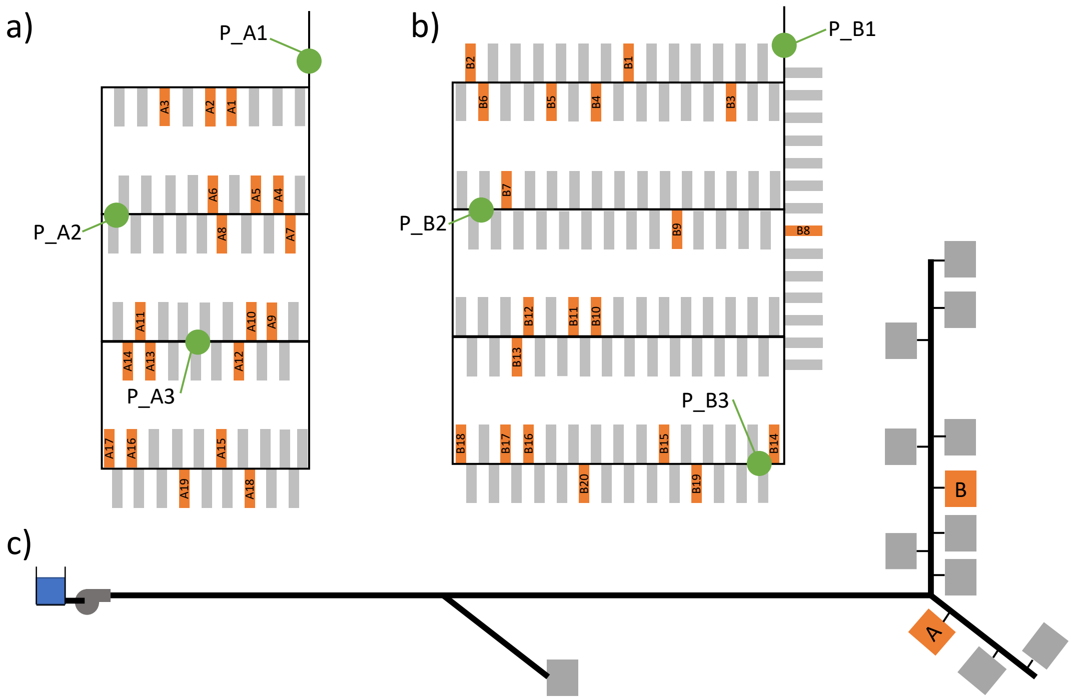

Fig. S2: Study site layout. a) An approximate layout of the Control Zone. b) An approximate layout of the Impact Zone. In both zones, approximate grab sampling locations are highlighted in orange and pressure loggers are labelled in green. c) The location of both Control (A) and Impact (B) zones with respect to the supply reservoir and other zones. Spatial layouts are representative, but not to scale.

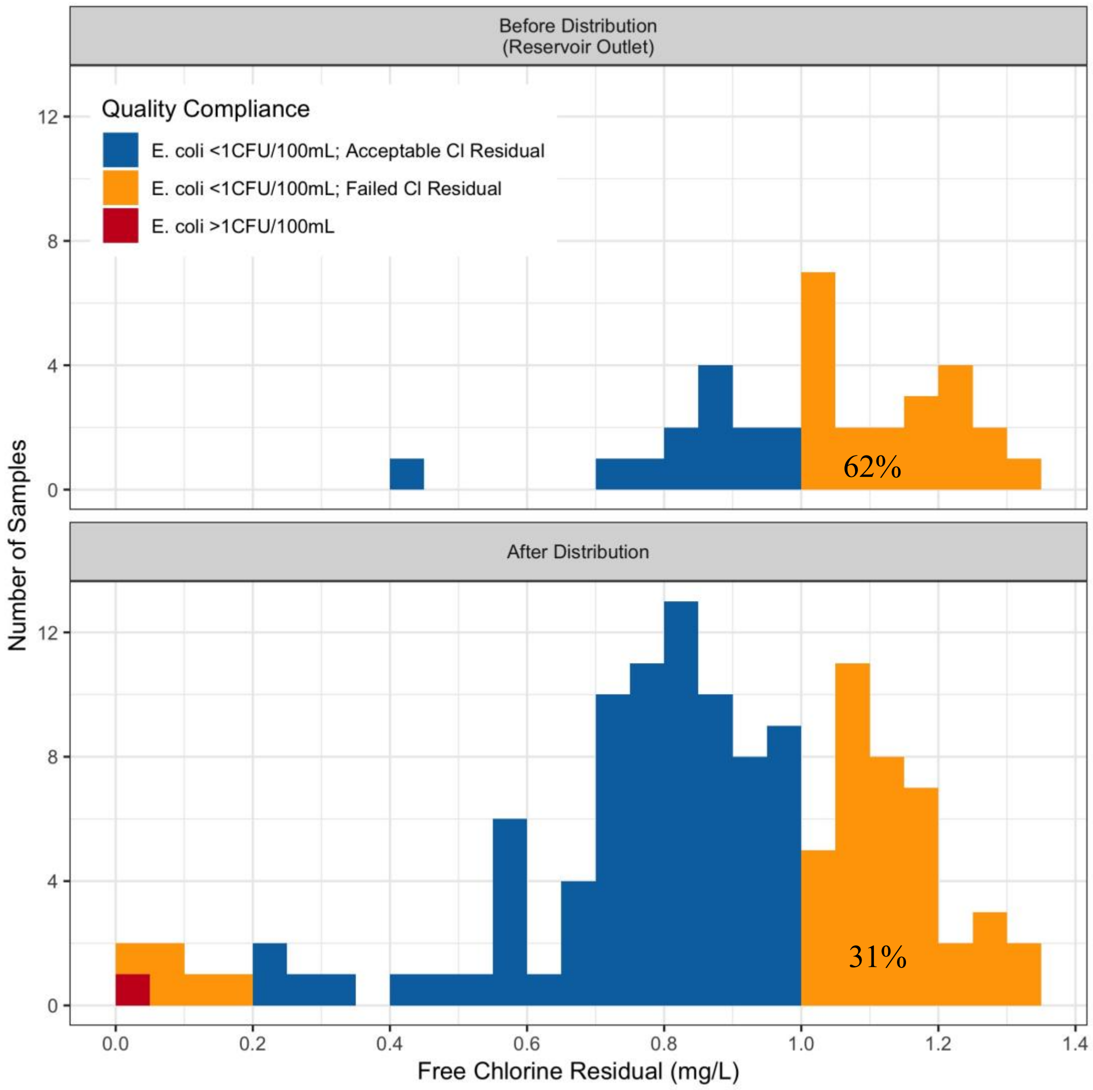

Fig. S3. Pre-study sampling of free chlorine and *E. coli*. Histograms of measured residual free chlorine are shaded based on Indian guidelines, which target samples between 0.2 and 1 mg/L (Bureau of Indian Standards, 2012). Blue indicates samples within the targets, yellow outside the targets. Red shading indicates a sample in which *E. coli* was detected. Top panel summarizes samples taken at the reservoir outlet; bottom panel summarizes samples from the distribution network.

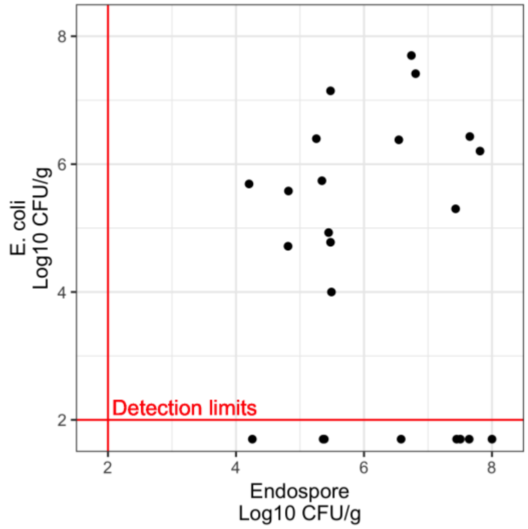

Fig. S4. Concentration and correlation of aerobic endospores and *E. coli* in sampled soil. Detection limits were 100 CFU/g of soil for both species. Dots below the detection limit indicate non-detection.

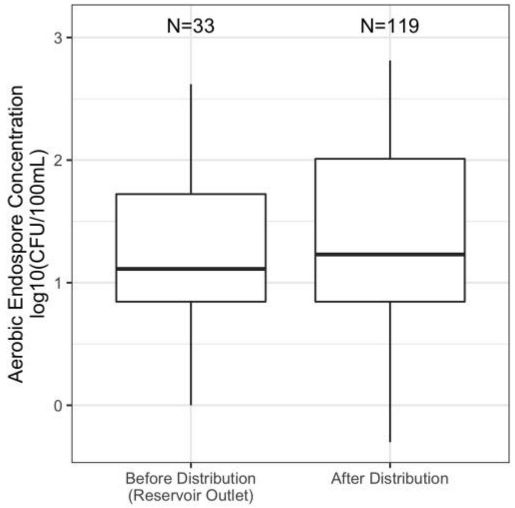

Figure S5: Aerobic endospore concentrations in treated water before and after distribution. Varied presence of aerobic endospores in the supply reservoir (before distribution) prevented their use as an intrusion indicator.

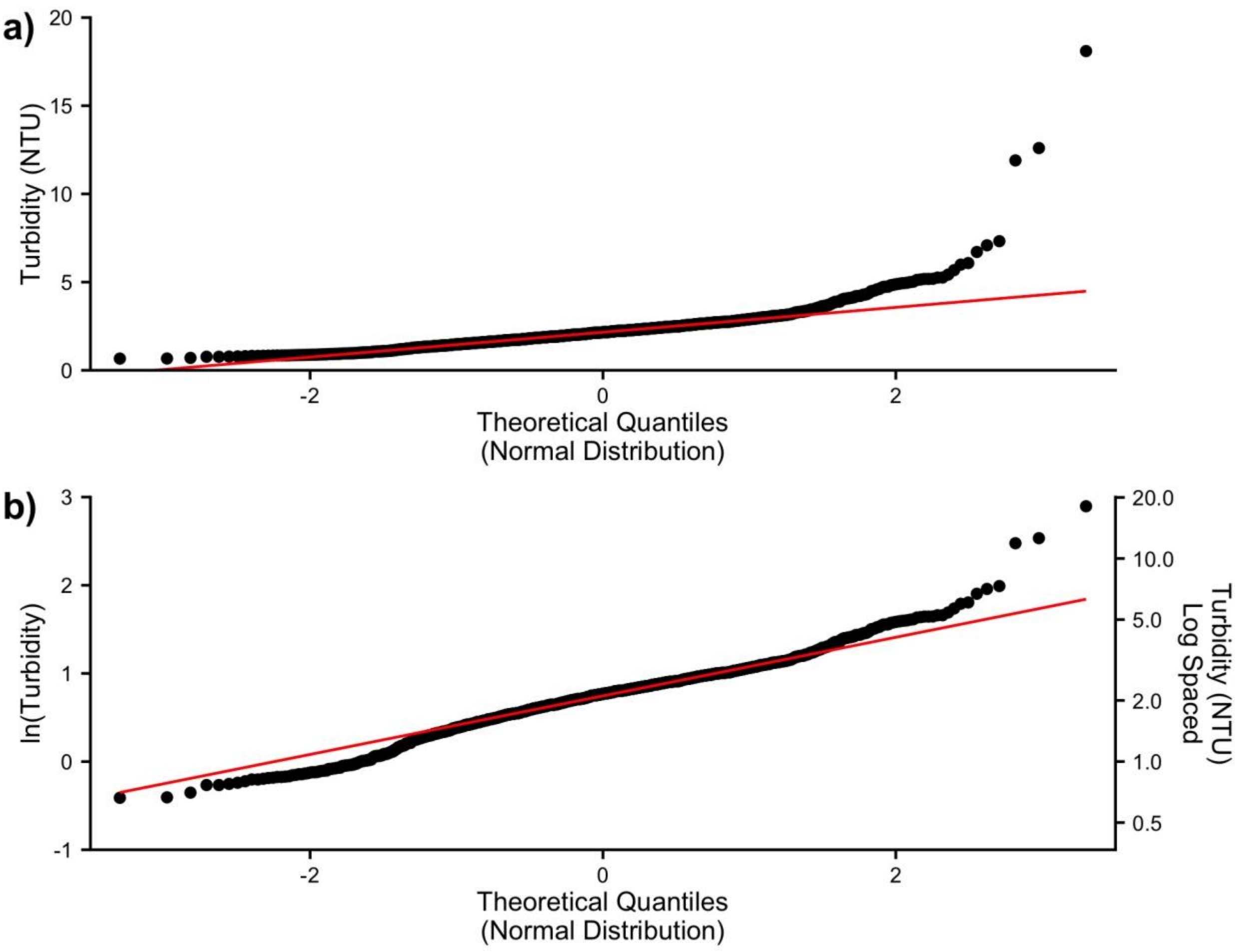

Fig. S6. Distribution of turbidity and log-turbidity values. Comparing the distribution of measured turbidity (a) and log-turbidity (b) values to the theoretically expected quantiles for normally distributed data. Log-transforming turbidity values reduced the right-skew.

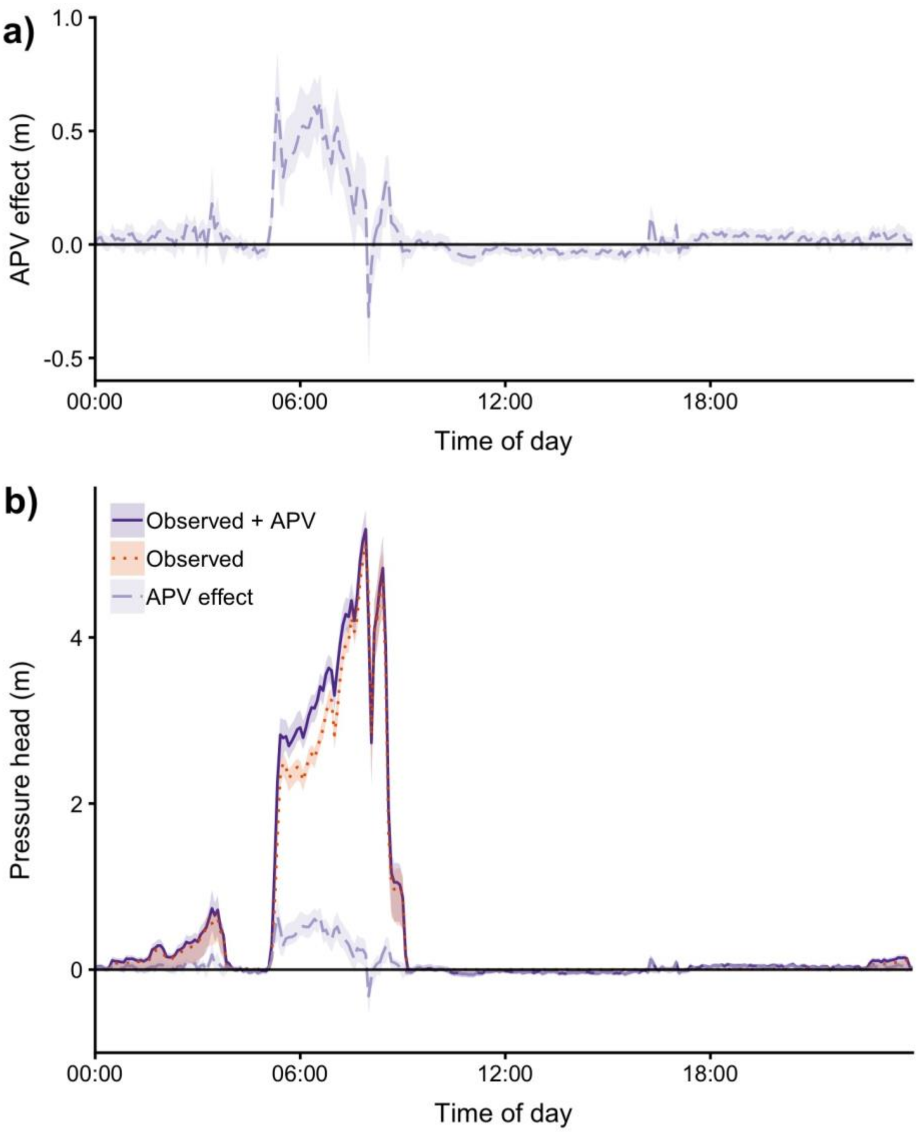

Fig. S7: Estimated effect APVs would have had in the Control Zone at all times. a) The regression-estimated (Model 3) APV effect at different times throughout the day. b) The effect APVs would have had on the Control Zone in the After Period (solid purple line; Observed + APV) was estimated as the superposition of observed mean pressures in the Control Zone during the After Period (dotted orange line; Observed) and the estimated effects of APVs (dashed light purple line; APV effect). Shaded bands represent 95% bias-corrected, accelerated confidence intervals. APV: Anti-Pump Valve.

Table S1. Prevalence of consumer pumps in intermittent water supplies. Pooled estimate is weighted by sample size.

**Source**	**Location**	**Survey year**	**Sample size**	**Consumer pump**	
				All pumps	Su
(Mastaller and Klingel, 2018)	Tiruvannamalai, India	2014	715	70%	
(Kumpel et al., 2017)	Hubli-Dharwad, India	2010-11	1951	58%	
(Onda, 2014)	Nagpur and Amravati, India	2013	41	25%	
(Klingel, 2010)	Béni Abbès, Algeria	2005	407	41%	
(Ahmed, 2008)	Karachi, Pakistan	2003	470		
Pooled Estimate			3594	58%	

Table S2. Full water quality regression models (Models 1,2 and S1).

Effect Estimates & Goodness of Fit	Dependent variable		
	ln(Turbidity) Model 1	Total Chlorine (mg/L) Model S1	Free Chlorine (mg/L) Model 2
Constant (b_0)	0.96*** (0.89, 1.04)	1.77*** (1.71, 1.84)	1.56*** (1.50, 1.62)
APV (b_1)	-0.08** (-0.13, -0.02)	0.04* (0.01, 0.07)	0.05*** (0.02, 0.09)
Turbidity (b_2)		-0.03*** (-0.05, -0.02)	-0.03** (-0.04, -0.02)
DATES:			
10/3/18	-0.02 (-0.13, 0.09)	-0.56*** (-0.61, -0.50)	-0.46*** (-0.52, -0.40)
10/4/18	0.08 (-0.10, 0.24)	-0.50*** (-0.61, -0.43)	-0.41*** (-0.52, -0.34)
10/8/18	-0.08 (-0.19, 0.03)	-0.32*** (-0.47, -0.22)	-0.27*** (-0.42, -0.15)
10/9/18	0.07 (-0.07, 0.20)	-0.61*** (-0.72, -0.54)	-0.58*** (-0.72, -0.47)
10/10/18	0.11 (-0.04, 0.39)	-0.44*** (-0.50, -0.38)	-0.33*** (-0.40, -0.26)
10/13/18	-0.23** (-0.32, -0.09)	-0.70*** (-0.76, -0.65)	-0.62*** (-0.68, -0.57)
10/14/18	-0.38*** (-0.52, -0.22)	-0.55*** (-0.62, -0.49)	-0.48*** (-0.56, -0.42)
10/20/18	-0.13** (-0.20, -0.05)	-0.68*** (-0.73, -0.62)	-0.68*** (-0.74, -0.63)
10/22/18	-0.78*** (-0.89, -0.69)	-0.46*** (-0.53, -0.39)	-0.51*** (-0.64, -0.42)
10/23/18	-0.84*** (-0.99, -0.66)	-0.70*** (-0.79, -0.62)	-0.76*** (-0.87, -0.67)
10/24/18	-0.94*** (-1.07, -0.77)	-0.52*** (-0.70, -0.41)	-0.52*** (-0.69, -0.42)
10/25/18	-0.88*** (-1.02, -0.47)	-0.84*** (-0.92, -0.77)	-0.87*** (-0.95, -0.80)
10/27/18	-1.08*** (-1.18, -0.98)	-0.52*** (-0.58, -0.46)	-0.60*** (-0.67, -0.54)
10/29/18	-0.57*** (-0.70, -0.44)	-0.81*** (-0.89, -0.74)	-0.86*** (-0.95, -0.78)
10/30/18	-0.43*** (-0.54, -0.34)	-0.89*** (-0.98, -0.82)	-0.92*** (-1.00, -0.85)
10/31/18	-0.28*** (-0.45, -0.16)	-0.71*** (-0.86, -0.60)	-0.79*** (-0.93, -0.68)
11/1/18	-0.26** (-0.39, -0.08)	-0.72*** (-0.85, -0.63)	-0.75*** (-0.86, -0.67)
11/3/18	-0.19*** (-0.40, -0.09)	-0.81*** (-0.86, -0.75)	-0.87*** (-0.93, -0.81)
11/5/18	-0.13* (-0.29, -0.01)	-1.09*** (-1.20, -1.01)	-1.19*** (-1.27, -1.11)
11/6/18	0.07* (0.002, 0.13)	-0.86*** (-0.91, -0.81)	-1.01*** (-1.06, -0.96)
11/8/18	0.07* (0.002, 0.15)	-0.74*** (-0.78, -0.68)	-0.84*** (-0.89, -0.79)
11/10/18	-0.07* (-0.15, -0.01)	-0.58*** (-0.63, -0.53)	-0.65*** (-0.70, -0.59)
11/12/18	-0.13* (-0.26, -0.02)	-0.90*** (-1.00, -0.82)	-0.94*** (-1.04, -0.87)
11/13/18	-0.26*** (-0.35, -0.18)	-0.74*** (-0.80, -0.68)	-0.72*** (-0.78, -0.66)
11/15/18	-0.32*** (-0.40, -0.24)	-0.75*** (-0.80, -0.69)	-0.72*** (-0.78, -0.66)
11/17/18	-0.24*** (-0.42, -0.11)	-0.36*** (-0.53, -0.25)	-0.32*** (-0.49, -0.21)
11/19/18	-0.13* (-0.27, -0.01)	-0.15*** (-0.21, -0.09)	-0.08* (-0.15, -0.01)
12/3/18	0.19** (0.05, 0.33)	-0.47*** (-0.55, -0.40)	-0.41*** (-0.50, -0.32)
12/4/18	0.33*** (0.23, 0.43)	-0.39*** (-0.45, -0.34)	-0.30*** (-0.36, -0.24)
12/5/18	0.30** (0.11, 0.48)	-0.64*** (-0.71, -0.57)	-0.55*** (-0.63, -0.48)
12/6/18	0.18*** (0.08, 0.33)	-0.38*** (-0.44, -0.31)	-0.31*** (-0.36, -0.26)
12/7/18	0.26*** (0.12, 0.38)	-0.43*** (-0.51, -0.36)	-0.38*** (-0.47, -0.30)
12/11/18	-0.17*** (-0.25, -0.09)	-0.30*** (-0.37, -0.23)	-0.24*** (-0.31, -0.16)

12/13/18	-0.18* (-0.26, -0.03)	-0.22*** (-0.27, -0.17)	-0.13*** (-0.19, -0.08)
12/14/18	-0.07 (-0.21, 0.05)	-0.33*** (-0.40, -0.27)	-0.26*** (-0.33, -0.19)
12/17/18	-0.06 (-0.18, 0.05)	-0.37*** (-0.43, -0.30)	-0.30*** (-0.37, -0.23)
12/18/18	-0.02 (-0.08, 0.04)	-0.33*** (-0.37, -0.28)	-0.27*** (-0.32, -0.23)
12/19/18	0.14* (0.01, 0.23)	-0.38*** (-0.45, -0.29)	-0.31*** (-0.39, -0.24)
12/24/18	-0.31*** (-0.45, -0.21)	-0.47*** (-0.54, -0.40)	-0.43*** (-0.51, -0.36)
12/26/18	-0.60*** (-0.73, -0.50)	-0.39*** (-0.45, -0.32)	-0.31*** (-0.39, -0.24)
12/27/18	-0.60*** (-0.68, -0.51)	-0.32*** (-0.37, -0.27)	-0.24*** (-0.30, -0.20)
12/28/18	-0.54*** (-0.67, -0.44)	-0.34*** (-0.40, -0.27)	-0.28*** (-0.35, -0.21)
1/2/19	-0.13 (-0.39, 0.31)	-0.43*** (-0.50, -0.37)	-0.39*** (-0.47, -0.32)
1/3/19	0.52*** (0.40, 0.61)	-0.33*** (-0.41, -0.26)	-0.30*** (-0.38, -0.24)
1/4/19	-0.29*** (-0.42, -0.19)	-0.44*** (-0.50, -0.37)	-0.41*** (-0.48, -0.33)
1/7/19	-0.14** (-0.27, -0.04)	-0.42*** (-0.49, -0.36)	-0.39*** (-0.46, -0.32)
1/8/19	-0.30*** (-0.40, -0.19)	-0.38*** (-0.43, -0.33)	-0.33*** (-0.38, -0.28)
1/12/19	-0.28*** (-0.35, -0.22)	-0.56*** (-0.60, -0.51)	-0.49*** (-0.54, -0.44)
1/14/19	-0.34*** (-0.46, -0.24)	-0.28*** (-0.34, -0.21)	-0.25*** (-0.33, -0.18)
1/15/19	-0.38*** (-0.46, -0.28)	-0.25*** (-0.30, -0.21)	-0.23*** (-0.28, -0.18)
1/16/19	-0.54*** (-0.67, -0.44)	-0.35*** (-0.42, -0.28)	-0.34*** (-0.42, -0.27)
1/18/19	-0.29*** (-0.41, -0.18)	-0.61*** (-0.67, -0.54)	-0.56*** (-0.64, -0.49)
CONNECTIONS			
A2	-0.02 (-0.12, 0.11)	0.08* (0.01, 0.14)	0.08* (0.01, 0.15)
A3	0.05 (-0.03, 0.14)	0.03 (-0.01, 0.07)	0.04* (0.002, 0.09)
A4	0.08 (-0.01, 0.16)	0.04 (-0.003, 0.08)	0.05* (0.004, 0.09)
A5	0.08 (-0.02, 0.21)	0.08** (0.02, 0.14)	0.09** (0.03, 0.16)
A6	0.04 (-0.07, 0.18)	0.07* (0.01, 0.13)	0.06 (-0.004, 0.14)
A7	0.08 (-0.05, 0.24)	0.04 (-0.07, 0.12)	0.02 (-0.08, 0.11)
A8	0.04 (-0.04, 0.16)	0.03 (-0.02, 0.07)	0.04 (-0.01, 0.08)
A9	0.02 (-0.05, 0.12)	0.03 (-0.01, 0.07)	0.04* (0.01, 0.09)
A10	-0.02 (-0.13, 0.11)	0.09** (0.03, 0.15)	0.11*** (0.05, 0.18)
A11	0.03 (-0.09, 0.17)	0.09** (0.03, 0.15)	0.11*** (0.05, 0.18)
A12	-0.000 (-0.08, 0.09)	0.01 (-0.02, 0.05)	0.03 (-0.01, 0.07)
A13	0.05 (-0.06, 0.18)	0.09** (0.03, 0.15)	0.11** (0.04, 0.18)
A14	-0.02 (-0.16, 0.07)	0.03 (-0.01, 0.07)	0.04* (0.01, 0.08)
A15	-0.02 (-0.14, 0.12)	0.05 (-0.02, 0.12)	0.08* (0.01, 0.14)
A16	-0.03 (-0.12, 0.09)	-0.04 (-0.09, 0.01)	-0.02 (-0.07, 0.03)
A17	-0.01 (-0.14, 0.15)	0.03 (-0.03, 0.10)	0.05 (-0.02, 0.12)
A18	-0.01 (-0.10, 0.08)	-0.01 (-0.06, 0.04)	0.003 (-0.05, 0.05)
A19	-0.01 (-0.13, 0.13)	-0.03 (-0.08, 0.01)	-0.01 (-0.06, 0.03)
B1	0.08 (-0.01, 0.22)	-0.10** (-0.20, -0.03)	-0.10** (-0.18, -0.03)
B2	0.06 (-0.06, 0.19)	-0.08 (-0.19, 0.01)	-0.12** (-0.21, -0.03)
B3	0.08 (-0.03, 0.22)	-0.05 (-0.15, 0.03)	-0.03 (-0.12, 0.05)
B4	0.02 (-0.10, 0.12)	-0.10** (-0.20, -0.03)	-0.16*** (-0.26, -0.08)
B5	-0.01 (-0.14, 0.12)	0.01 (-0.08, 0.07)	-0.03 (-0.12, 0.03)

B6	0.02 (-0.10, 0.12)	-0.08* (-0.18, -0.02)	-0.11** (-0.20, -0.04)
B7	-0.07 (-0.21, 0.02)	-0.06* (-0.17, -0.001)	-0.09*** (-0.20, -0.03)
B8	0.002 (-0.08, 0.08)	-0.01 (-0.11, 0.05)	-0.07* (-0.17, -0.01)
B9	-0.04 (-0.17, 0.09)	0.02 (-0.06, 0.08)	0.004 (-0.07, 0.07)
B10	-0.001 (-0.11, 0.12)	0.04 (-0.01, 0.10)	0.000 (-0.06, 0.06)
B11	-0.05 (-0.13, 0.04)	-0.01 (-0.07, 0.04)	-0.13*** (-0.23, -0.05)
B12	-0.01 (-0.09, 0.07)	0.02 (-0.03, 0.08)	-0.05* (-0.10, -0.005)
B13	0.01 (-0.11, 0.22)	0.05 (-0.01, 0.13)	-0.03 (-0.10, 0.04)
B14	0.06 (-0.07, 0.33)	0.02 (-0.04, 0.07)	-0.04 (-0.13, 0.03)
B15	-0.01 (-0.10, 0.07)	-0.04 (-0.09, 0.01)	-0.10*** (-0.18, -0.04)
B16	-0.07 (-0.17, 0.06)	0.01 (-0.08, 0.07)	-0.06 (-0.14, 0.01)
B17	-0.03 (-0.14, 0.06)	-0.02 (-0.09, 0.04)	-0.08** (-0.15, -0.02)
B18	-0.04 (-0.15, 0.10)	0.03 (-0.04, 0.10)	-0.03 (-0.10, 0.03)
B19	0.09 (-0.09, 0.48)	0.02 (-0.03, 0.08)	-0.02 (-0.08, 0.05)
B20	0.000 (-0.09, 0.15)	-0.06* (-0.16, -0.004)	-0.10*** (-0.17, -0.05)
Observations	1,031	1,031	1,031
Adjusted R^2	0.66	0.79	0.83
F Statistic	22*** (df = 91; 939)	43*** (df = 92; 938)	57*** (df = 92; 938)

Notes: $^{*}p<0.05$; $^{**}p<0.01$; $^{***}p<0.001$; bracketed terms following effect estimates are the 95% bias-corrected, accelerated percentile intervals for each effect estimate. Turbidity is measured in NTU. Regression Models 1, 2, and 3 estimate ln(turbidity), total chlorine, and free chlorine, respectively. df: degrees of freedom; APV: Anti-Pump Valve.

Table S3: Regression Model 3, estimating APV effects on pressure by time of day.

Coefficient	Estimate
Constant (b_0)	0.09*** (0.06, 0.11)
APV at 00:00	0.01 (-0.08, 0.09)
APV at 00:05	0.03 (-0.06, 0.11)
APV at 00:10	0.03 (-0.05, 0.12)
APV at 00:15	0.01 (-0.07, 0.09)
APV at 00:20	0.002 (-0.08, 0.09)
APV at 00:25	0.01 (-0.08, 0.09)
APV at 00:30	0.05* (-0.04, 0.13)
APV at 00:35	0.02 (-0.06, 0.11)
APV at 00:40	0.03 (-0.06, 0.11)
APV at 00:45	0.02 (-0.06, 0.11)
APV at 00:50	0.02 (-0.07, 0.10)
APV at 00:55	0.05 (-0.03, 0.14)
APV at 01:00	0.04 (-0.04, 0.12)
APV at 01:05	0.05* (-0.03, 0.14)
APV at 01:10	0.02 (-0.06, 0.11)
APV at 01:15	0.01 (-0.07, 0.10)
APV at 01:20	0.01 (-0.07, 0.10)
APV at 01:25	-0.002 (-0.09, 0.08)
APV at 01:30	0.01 (-0.07, 0.10)
APV at 01:35	0.02 (-0.06, 0.11)
APV at 01:40	0.04 (-0.05, 0.12)
APV at 01:45	0.03 (-0.05, 0.11)
APV at 01:50	0.06 (-0.03, 0.14)
APV at 01:55	0.06 (-0.03, 0.14)
APV at 02:00	0.05 (-0.04, 0.13)
APV at 02:05	0.03 (-0.05, 0.12)
APV at 02:10	0.02 (-0.06, 0.11)
APV at 02:15	0.02 (-0.06, 0.10)
APV at 02:20	-0.01 (-0.09, 0.08)
APV at 02:25	0.04 (-0.04, 0.12)
APV at 02:30	0.04 (-0.04, 0.13)
APV at 02:35	0.02 (-0.06, 0.11)
APV at 02:40	0.08* (-0.0004, 0.17)
APV at 02:45	0.06 (-0.03, 0.14)
APV at 02:50	0.05 (-0.03, 0.13)
APV at 02:55	0.08* (-0.01, 0.16)
APV at 03:00	0.002 (-0.08, 0.09)
APV at 03:05	0.05 (-0.04, 0.13)
APV at 03:10	0.06 (-0.03, 0.14)
APV at 03:15	-0.02 (-0.10, 0.06)
APV at 03:20	0.07* (-0.01, 0.16)
APV at 03:25	0.20* (0.11, 0.28)
APV at 03:30	0.07 (-0.01, 0.16)
APV at 03:35	0.09 (0.01, 0.18)
APV at 03:40	0.04 (-0.05, 0.12)
APV at 03:45	0.003 (-0.08, 0.09)
APV at 03:50	0.03 (-0.06, 0.11)
APV at 03:55	0.03 (-0.05, 0.11)
APV at 04:00	0.01 (-0.07, 0.10)
APV at 04:05	-0.003 (-0.09, 0.08)
APV at 04:10	-0.01 (-0.09, 0.07)
APV at 04:15	0.01 (-0.08, 0.09)
APV at 04:20	-0.02 (-0.10, 0.06)
APV at 04:25	-0.01 (-0.09, 0.07)
APV at 04:30	-0.03 (-0.12, 0.05)
APV at 04:35	-0.03 (-0.11, 0.05)
APV at 04:40	-0.01 (-0.10, 0.07)
APV at 04:45	-0.04* (-0.12, 0.04)
APV at 04:50	-0.03 (-0.11, 0.05)
APV at 04:55	-0.02 (-0.11, 0.06)
APV at 05:00	-0.03 (-0.11, 0.05)
APV at 05:05	0.03 (-0.06, 0.11)
APV at 05:10	0.13** (0.05, 0.21)
APV at 05:15	0.46*** (0.38, 0.54)
APV at 05:20	0.62*** (0.54, 0.71)
APV at 05:25	0.44** (0.36, 0.53)
APV at 05:30	0.31*** (0.22, 0.39)
APV at 05:35	0.39** (0.31, 0.47)
APV at 05:40	0.41** (0.32, 0.49)
APV at 05:45	0.42** (0.33, 0.50)
APV at 05:50	0.43** (0.35, 0.52)
APV at 05:55	0.48** (0.39, 0.56)
APV at 06:00	0.53** (0.45, 0.62)
APV at 06:05	0.53*** (0.45, 0.62)
APV at 06:10	0.52*** (0.43, 0.60)
APV at 06:15	0.51*** (0.43, 0.60)
APV at 06:20	0.56*** (0.47, 0.64)
APV at 06:25	0.61*** (0.52, 0.69)
APV at 06:30	0.57*** (0.49, 0.66)
APV at 06:35	0.63*** (0.54, 0.71)
APV at 06:40	0.46*** (0.38, 0.55)
APV at 06:45	0.48*** (0.39, 0.56)
APV at 06:50	0.40*** (0.32, 0.49)
APV at 06:55	0.35*** (0.26, 0.43)

APV at 07:00	0.47*** (0.39, 0.56)
APV at 07:05	0.52*** (0.44, 0.61)
APV at 07:10	0.42*** (0.34, 0.51)
APV at 07:15	0.40*** (0.32, 0.48)
APV at 07:20	0.35*** (0.26, 0.43)
APV at 07:25	0.30*** (0.21, 0.38)
APV at 07:30	0.21* (0.12, 0.29)
APV at 07:35	0.14 (0.05, 0.22)
APV at 07:40	0.25** (0.17, 0.34)
APV at 07:45	0.23** (0.15, 0.32)
APV at 07:50	0.23* (0.15, 0.31)
APV at 07:55	0.18* (0.10, 0.26)
APV at 08:00	-0.32** (-0.40, -0.24)
APV at 08:05	-0.09 (-0.17, -0.004)
APV at 08:10	0.01 (-0.08, 0.09)
APV at 08:15	0.06 (-0.02, 0.15)
APV at 08:20	0.07 (-0.01, 0.16)
APV at 08:25	0.19** (0.10, 0.27)
APV at 08:30	0.25** (0.17, 0.34)
APV at 08:35	0.28*** (0.20, 0.36)
APV at 08:40	0.09 (0.01, 0.17)
APV at 08:45	0.07 (-0.01, 0.16)
APV at 08:50	0.06 (-0.02, 0.14)
APV at 08:55	0.03 (-0.05, 0.11)
APV at 09:00	-0.02 (-0.10, 0.06)
APV at 09:05	-0.02 (-0.10, 0.06)
APV at 09:10	-0.02 (-0.10, 0.07)
APV at 09:15	-0.03 (-0.11, 0.06)
APV at 09:20	-0.005 (-0.09, 0.08)
APV at 09:25	0.02 (-0.06, 0.11)
APV at 09:30	0.02 (-0.06, 0.11)
APV at 09:35	0.01 (-0.08, 0.09)
APV at 09:40	0.01 (-0.07, 0.10)
APV at 09:45	0.02 (-0.07, 0.10)
APV at 09:50	0.02 (-0.06, 0.10)
APV at 09:55	-0.003 (-0.09, 0.08)
APV at 10:00	0.005 (-0.08, 0.09)
APV at 10:05	0.01 (-0.07, 0.10)
APV at 10:10	0.01 (-0.07, 0.09)
APV at 10:15	-0.002 (-0.09, 0.08)
APV at 10:20	-0.01 (-0.10, 0.07)
APV at 10:25	-0.03 (-0.12, 0.05)
APV at 10:30	-0.04 (-0.12, 0.04)
APV at 10:35	-0.04 (-0.12, 0.05)
APV at 10:40	-0.04 (-0.12, 0.04)
APV at 10:45	-0.05* (-0.13, 0.04)
APV at 10:50	-0.05* (-0.13, 0.04)
APV at 10:55	-0.06** (-0.14, 0.03)
APV at 11:00	-0.06* (-0.14, 0.03)
APV at 11:05	-0.06* (-0.14, 0.03)
APV at 11:10	-0.04 (-0.12, 0.05)
APV at 11:15	-0.04* (-0.13, 0.04)
APV at 11:20	-0.04* (-0.12, 0.04)
APV at 11:25	-0.02 (-0.11, 0.06)
APV at 11:30	-0.02 (-0.11, 0.06)
APV at 11:35	-0.02 (-0.11, 0.06)
APV at 11:40	-0.02 (-0.10, 0.07)
APV at 11:45	-0.01 (-0.10, 0.07)
APV at 11:50	-0.02 (-0.11, 0.06)
APV at 11:55	-0.01 (-0.10, 0.07)
APV at 12:00	-0.02 (-0.10, 0.07)
APV at 12:05	-0.02 (-0.10, 0.07)
APV at 12:10	-0.03* (-0.11, 0.06)
APV at 12:15	-0.03 (-0.11, 0.06)
APV at 12:20	-0.03* (-0.11, 0.05)
APV at 12:25	-0.02 (-0.10, 0.06)
APV at 12:30	-0.04*** (-0.12, 0.04)
APV at 12:35	-0.03* (-0.12, 0.05)
APV at 12:40	-0.03* (-0.11, 0.06)
APV at 12:45	-0.03* (-0.11, 0.06)
APV at 12:50	-0.04** (-0.12, 0.04)
APV at 12:55	-0.03* (-0.11, 0.06)
APV at 13:00	-0.02* (-0.11, 0.06)
APV at 13:05	-0.05*** (-0.13, 0.04)
APV at 13:10	-0.04** (-0.12, 0.05)
APV at 13:15	-0.02 (-0.11, 0.06)
APV at 13:20	-0.03* (-0.11, 0.06)
APV at 13:25	-0.02* (-0.11, 0.06)
APV at 13:30	-0.03** (-0.11, 0.05)
APV at 13:35	-0.05*** (-0.13, 0.04)
APV at 13:40	-0.04*** (-0.12, 0.04)
APV at 13:45	-0.05*** (-0.13, 0.03)
APV at 13:50	-0.04** (-0.12, 0.05)
APV at 13:55	-0.04** (-0.12, 0.05)
APV at 14:00	-0.03** (-0.12, 0.05)
APV at 14:05	-0.04*** (-0.12, 0.04)
APV at 14:10	-0.03* (-0.12, 0.05)
APV at 14:15	-0.03* (-0.11, 0.06)
APV at 14:20	-0.03** (-0.12, 0.05)
APV at 14:25	-0.03* (-0.11, 0.05)

APV at 14:30	-0.04*** (-0.12, 0.04)	APV at 17:00	0.08 (-0.01, 0.16)	APV at 19:30	0.04* (-0.05, 0.12)
APV at 14:35	-0.04*** (-0.13, 0.04)	APV at 17:05	-0.03 (-0.12, 0.05)	APV at 19:35	0.04* (-0.05, 0.12)
APV at 14:40	-0.04*** (-0.13, 0.04)	APV at 17:10	-0.01 (-0.10, 0.07)	APV at 19:40	0.02 (-0.06, 0.10)
APV at 14:45	-0.04*** (-0.13, 0.04)	APV at 17:15	-0.01 (-0.10, 0.07)	APV at 19:45	0.03 (-0.06, 0.11)
APV at 14:50	-0.03** (-0.11, 0.06)	APV at 17:20	-0.01 (-0.10, 0.07)	APV at 19:50	0.04* (-0.04, 0.12)
APV at 14:55	-0.03** (-0.11, 0.05)	APV at 17:25	0.01 (-0.07, 0.09)	APV at 19:55	0.04** (-0.04, 0.13)
APV at 15:00	-0.04** (-0.12, 0.05)	APV at 17:30	0.02 (-0.06, 0.11)	APV at 20:00	0.03* (-0.05, 0.12)
APV at 15:05	-0.03** (-0.11, 0.05)	APV at 17:35	0.02 (-0.07, 0.10)	APV at 20:05	0.04** (-0.04, 0.12)
APV at 15:10	-0.04*** (-0.13, 0.04)	APV at 17:40	0.02 (-0.06, 0.10)	APV at 20:10	0.02 (-0.06, 0.11)
APV at 15:15	-0.04** (-0.13, 0.04)	APV at 17:45	0.04** (-0.04, 0.13)	APV at 20:15	0.04* (-0.05, 0.12)
APV at 15:20	-0.05*** (-0.13, 0.04)	APV at 17:50	0.04* (-0.05, 0.12)	APV at 20:20	0.05*** (-0.04, 0.13)
APV at 15:25	-0.03** (-0.12, 0.05)	APV at 17:55	0.02 (-0.06, 0.11)	APV at 20:25	0.05** (-0.03, 0.13)
APV at 15:30	-0.06** (-0.14, 0.03)	APV at 18:00	0.02 (-0.06, 0.11)	APV at 20:30	0.02 (-0.06, 0.11)
APV at 15:35	-0.04** (-0.12, 0.04)	APV at 18:05	0.05** (-0.04, 0.13)	APV at 20:35	0.02 (-0.07, 0.10)
APV at 15:40	-0.03** (-0.12, 0.05)	APV at 18:10	0.04** (-0.04, 0.13)	APV at 20:40	0.03* (-0.05, 0.12)
APV at 15:45	-0.02* (-0.10, 0.06)	APV at 18:15	0.04** (-0.04, 0.13)	APV at 20:45	0.03* (-0.05, 0.12)
APV at 15:50	-0.02 (-0.10, 0.07)	APV at 18:20	0.05** (-0.03, 0.13)	APV at 20:50	0.02 (-0.06, 0.10)
APV at 15:55	-0.04** (-0.12, 0.05)	APV at 18:25	0.03* (-0.05, 0.12)	APV at 20:55	0.02 (-0.06, 0.10)
APV at 16:00	-0.02 (-0.10, 0.07)	APV at 18:30	0.05** (-0.04, 0.13)	APV at 21:00	0.04* (-0.05, 0.12)
APV at 16:05	-0.01 (-0.09, 0.07)	APV at 18:35	0.05** (-0.03, 0.13)	APV at 21:05	0.02 (-0.07, 0.10)
APV at 16:10	-0.03** (-0.11, 0.05)	APV at 18:40	0.03* (-0.05, 0.12)	APV at 21:10	0.003 (-0.08, 0.09)
APV at 16:15	0.10 (0.02, 0.18)	APV at 18:45	0.04* (-0.05, 0.12)	APV at 21:15	0.02 (-0.06, 0.11)
APV at 16:20	0.06 (-0.02, 0.14)	APV at 18:50	0.03 (-0.05, 0.12)	APV at 21:20	0.03* (-0.05, 0.11)
APV at 16:25	-0.001 (-0.09, 0.08)	APV at 18:55	0.04 (-0.05, 0.12)	APV at 21:25	0.02 (-0.06, 0.11)
APV at 16:30	-0.01 (-0.10, 0.07)	APV at 19:00	0.05** (-0.04, 0.13)	APV at 21:30	-0.01 (-0.09, 0.08)
APV at 16:35	0.03 (-0.05, 0.12)	APV at 19:05	0.03* (-0.05, 0.12)	APV at 21:35	0.02 (-0.06, 0.10)
APV at 16:40	0.005 (-0.08, 0.09)	APV at 19:10	0.05** (-0.04, 0.13)	APV at 21:40	0.02 (-0.06, 0.11)
APV at 16:45	0.002 (-0.08, 0.09)	APV at 19:15	0.04** (-0.04, 0.12)	APV at 21:45	0.04* (-0.04, 0.12)
APV at 16:50	-0.001 (-0.08, 0.08)	APV at 19:20	0.03 (-0.05, 0.11)	APV at 21:50	0.01 (-0.07, 0.10)
APV at 16:55	0.004 (-0.08, 0.09)	APV at 19:25	0.04** (-0.05, 0.12)	APV at 21:55	0.02 (-0.07, 0.10)

APV at 22:00	0.03* (-0.05, 0.11)
APV at 22:05	0.03 (-0.06, 0.11)
APV at 22:10	0.01 (-0.07, 0.10)
APV at 22:15	0.03 (-0.06, 0.11)
APV at 22:20	0.03 (-0.05, 0.11)
APV at 22:25	-0.001 (-0.08, 0.08)
APV at 22:30	0.01 (-0.07, 0.09)
APV at 22:35	0.003 (-0.08, 0.09)
APV at 22:40	0.03 (-0.06, 0.11)
APV at 22:45	0.05 (-0.03, 0.13)
APV at 22:50	0.03 (-0.05, 0.11)
APV at 22:55	0.04 (-0.04, 0.13)
APV at 23:00	0.02 (-0.06, 0.11)
APV at 23:05	0.05 (-0.03, 0.14)
APV at 23:10	0.02 (-0.06, 0.11)
APV at 23:15	0.03 (-0.06, 0.11)
APV at 23:20	0.03 (-0.05, 0.12)
APV at 23:25	0.04 (-0.04, 0.13)
APV at 23:30	0.06 (-0.03, 0.14)
APV at 23:35	0.05 (-0.04, 0.13)
APV at 23:40	0.04 (-0.04, 0.13)
APV at 23:45	0.02 (-0.07, 0.10)
APV at 23:50	0.02 (-0.06, 0.10)
APV at 23:55	0.01 (-0.08, 0.09)
Time of Day ($b_{T_1,00:05} - b_{T_1,23:55}$)	df=278; F=47***
Observations	25,055
Adjusted R^2	0.40
F Statistic	30.12****** (df = 575; 24,479)

Notes: *p<0.05; **p<0.01; ***p<0.001; bracketed terms following effect estimates are the 95% bias-corrected, accelerated percentile intervals for each effect estimate. The significance of Time of Day factors is summarized by the F-Statistic (from Type II ANOVA). The APV effect estimates from regression Model 4 ($b_{T_2,00:00}, b_{T_2,00:05}, \ldots, b_{T_2,23:55}$) are described as (APV at 00:00, APV at 00:05, …, APV at 23:55. df: degrees of freedom; APV: Anti-Pump Valve.

Datasets

Dataset S2

Meyer, D., Slocum, A., 2020. Replication Data for: Taylor D (2014) Reducing booster-pump-induced contaminant intrusion in Indian water systems with a self-actuated, back-pressure regulating valve. https://doi.org/10.5683/SP2/FYIZNS

Dataset S3

Meyer, D., Whittle, A., Khari, J., Slocum, A., 2020. Baseline Data Supporting: Meyer D, Whittle A, Khari J, Slocum A (2021) Effects of hydraulically disconnecting consumer pumps in an intermittent water supply. v2.2. https://doi.org/10.5683/SP2/PGSTAH

References

Ahmed, N., 2008. Water Supply in Karachi: Issues and Prospects. Oxford University Press, Oxford, New York.

Altaf, M.A., 1994. Household demand for improved water and sanitation in a large secondary city: Findings from a study in Gujranwala, Pakistan. Habitat Int. 18, 45–55. https://doi.org/10.1016/0197-3975(94)90038-8

Cartier, C., Besner, M.C., Barbeau, B., 2009. Evaluating aerobic endospores as indicators of intrusion in distribution systems. J. Am. Water Works Assoc. 101, 46–58. https://doi.org/10.1002/j.1551-8833.2009.tb09923.x

Klingel, P., 2010. Von intermittierender zu kontinuierlicher Wasserverteilung in Entwicklungsländern (From intermittent to continuous water distribution in developing

countries) (Dr.-Ing. Dissertation). Institute for Water and River Basin Management, Karlsruhe Institute of Technology, Karlsruhe, Germany.

Kumpel, E., Woelfle-Erskine, C., Ray, I., Nelson, K.L., 2017. Measuring household consumption and waste in unmetered, intermittent piped water systems. Water Resour. Res. 53, 302–315. https://doi.org/10.1002/2016wr019702

Mastaller, M., Klingel, P., 2018. Application of a water balance adapted to intermittent water supply and flat-rate tariffs without customer metering in Tiruvannamalai, India. Water Sci. Technol. Water Supply 18, 347–356. https://doi.org/10.2166/ws.2017.121

Onda, K.S., 2014. Intermittent vs. Continuous Water Supply: What benefits do households actually receive? Evidence from two cities in India (Master's Report). University of North Carolina at Chapel Hill, Chapel Hill, USA.

Taylor, D., Slocum, A., 2020. Replication Data for: Taylor D (2014) Reducing booster-pump-induced contaminant intrusion in Indian water systems with a self-actuated, back-pressure regulating valve. https://doi.org/10.5683/SP2/FYIZNS

Taylor, D., Whittle, A., Khari, J., Slocum, A., 2020. Baseline Data Supporting: Taylor D, Whittle A, Khari J, Slocum A (2020) Consumer pumps in an intermittent water supply: the effects and feasibility of their removal. https://doi.org/10.5683/SP2/PGSTAH

Taylor, D.D.J., 2014. Reducing booster-pump-induced contaminant intrusion in Indian water systems with a self-actuated, back-pressure regulating valve (Thesis). Massachusetts Institute of Technology, Cambridge, MA.

Zérah, M.-H., 2000. Water, unreliable supply in Delhi. Manohar Publishers.
